# Mitochondrial Electron Flow Dynamics Imaging for Assessing Mitochondrial Quality and Drug Screening

**DOI:** 10.1002/advs.202410561

**Published:** 2025-01-13

**Authors:** Youxiao Ren, Ling‐Ling Wu, Wenjing Song, Yanan Gao, Litao Shao, Zhiyuan Lu, Songsong Wang, Xintian Shao, Zhenjie Yu, Mengrui Zhang, Jing Wu, Liwen Han, Kewu Zeng, Qixin Chen

**Affiliations:** ^1^ State Key Laboratory of Advanced Drug Delivery and Release Systems School of Pharmaceutical Sciences Medical Science and Technology Innovation Center Shandong First Medical University & Shandong Academy of Medical Sciences Jinan Shandong 250117 P. R. China; ^2^ Department of Traditional Chinese Medicine Orthopedics Neck‐Shoulder and Lumbocrural Pain Hospital Affiliated to Shandong First Medical University Jinan Shandong 250062 P. R. China; ^3^ Department of Pharmacy The First Affiliated Hospital of Shandong First Medical University & Shandong Provincial Qianfoshan Hospital Jinan 250014 P. R. China; ^4^ State Key Laboratory of Natural and Biomimetic Drugs School of Pharmaceutical Sciences Peking University Beijing 100191 P. R. China; ^5^ Departments of Diagnostic Radiology, Surgery Chemical and Biomolecular Engineering and Biomedical Engineering Yong Loo Lin School of Medicine and College of Design and Engineering National University of Singapore Singapore 119074 Singapore

**Keywords:** drug screening, imaging, mitochondria, mitochondrial electron flow, morphology

## Abstract

Mitochondrial quality control is paramount for cellular development, with mitochondrial electron flow (Mito‐EF) playing a central role in maintaining mitochondrial homeostasis. However, unlike visible protein entities, which can be monitored through chemical biotechnology, regulating mitochondrial quality control by invisible entities such as Mito‐EF has remained elusive. Here, a Mito‐EF tracker (Mito‐EFT) with a four‐pronged probe design is presented to elucidate the dynamic mechanisms of Mito‐EF's involvement in mitochondrial quality control. Heightened aggregation of Mito‐EF in fiber‐like healthy mitochondria compared to round‐like damaged mitochondria is demonstrated, revealed Mito‐EF aggregation correlated with mitochondrial morphological remodeling, particularly in regions undergoing mitochondrial fission and fusion, and show the Mito‐EF signal associated with mitochondrial cristae maintained by Dynamin‐Related Protein 1 (DRP1). This underscores the importance of considering Mito‐EF in assessing mitochondrial quality control parameters. A novel drug screening evaluation parameter, Mito‐EF is also introduced to screen and discover mitochondrial‐targeted therapeutic modulators. This tracker provides new avenues for investigating the role of Mito‐EF in maintaining mitochondrial homeostasis and quality control, offering a potent tool for assessing mitochondrial quality and drug screening.

## Graphical abstract

A Mito‐EF tracker (Mito‐EFT) is developed to investigate Mito‐EF's role in mitochondrial quality control. It maps the increased Mito‐EF dynamics in healthy, fiber‐like mitochondria compared to damaged, round ones and highlights its correlation with morphological remodeling during fission and fusion, thereby supporting Mito‐EF as a crucial parameter for screening mitochondrial‐targeted therapeutic modulators.

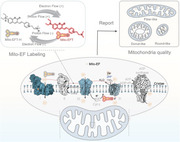

## 1 Introduction

Mitochondrial quality control is pivotal in metabolic diseases,^[^ ^1^ ^]^ with mitochondrial electron flow (Mito‐EF) as a crucial component in the respiratory chain to support energy supply.^[^ ^2^ ^]^ Mito‐EF involves electron transfer from nicotinamide adenine dinucleotide and reduced flavine adenine dinucleotide to cytochrome complexes,^[^ ^3^ ^]^ releasing energy to drive proton pumping from the mitochondrial matrix to the inner membrane space,^[^ ^4^ ^]^ thereby establishing a proton gradient.^[^ ^5^ ^]^ Proton flow utilizes this gradient to synthesize ATP, which is crucial for maintaining mitochondrial quality control.^[^ ^6^ ^]^ Consequently, mitochondrial electron flow and proton flow are mutually dependent processes.^[^ ^7^ ^]^ A recent study underscored the significance of manipulating Mito‐EF in cancer treatment.^[^ ^8^ ^]^ However, despite its presumed importance in regulating mitochondrial homeostasis and cell development, the direct role of Mito‐EF in mitochondrial quality control remains ambiguous due to the absence of tools specifically targeting it.

Developing a molecular probe tailored for Mito‐EF is challenging because electrons, being fundamental particles,^[^ ^9^ ^]^ lack chemical properties enabling direct participation in atomic bonding or ionic interactions. Instead, they act as observers or mediators in chemical reactions, essential for modulating molecular structure and properties,^[^ ^10^ ^]^ rendering probe monitoring challenging. Furthermore, Mito‐EF is indirectly reflected by the proton gradient in the inner mitochondrial membrane (IMM) region.^[^ ^11^ ^]^ The widely accepted understanding is that electron flow generates a proton gradient, resulting in an asymmetric distribution of protons across the mitochondrial membrane.^[^ ^12^ ^]^ Monitoring protons/electrons in the membrane region is susceptible to interference from the cytosol or other membrane structures, necessitating probes with high transmembrane and targeting capabilities. Moreover, Mito‐EF detection necessitates reversibility, as electrons/protons are indispensable for oxidative phosphorylation, and any interference can disrupt the electron transport chain, leading to proton leakage.^[^ ^4^ ^]^ Therefore, test probes must release electrons/protons after binding. Although IMM protons can be considered proxies for electron movement,^[^ ^13^ ^]^ currently available proton probes (also termed pH probes^[^ ^14^ ^]^) are typically localized within organelle matrixes, such as lysosomes^[^ ^15^ ^]^ and mitochondria,^[^ ^16^ ^]^ limiting their application. The absence of a specific probe for mitochondrial inner membrane electrons/protons impedes functional studies of Mito‐EF in mitochondrial homeostasis and development.

**Figure 1:**
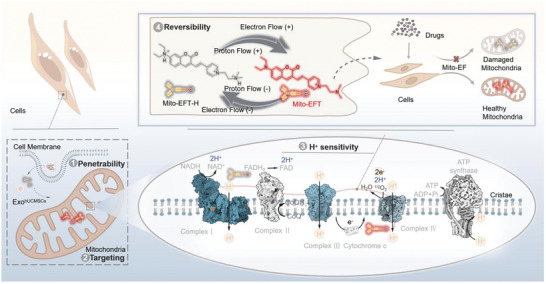
Schematic of the electronic flow‐responded mitochondrial fluorescent probe. Schematic representation of electronic flow‐responded mitochondrial fluorescent probe Mito‐EFT. In the mitochondrial electron transport chain, the initiation of electron flow induced the pumping of protons from the mitochondrial matrix into the mitochondrial intermembrane space, thereby establishing a correlated proton flow. The synchronization and positive correlation between electron flow and proton flow are notable. Considering the inherent challenges in directly monitoring the electron flow, the dynamic nuances of electron flow were assessed by observing alterations in the mitochondrial proton flow. This design includes 1) the designed probe Mito‐EFT encapsulated in exosomes to enhance transmembrane delivery and minimize fluorescence interference from the plasma membrane; 2) positive charges that target the IMM, ensuring the probe's precise localization; 3) sensitivity to the presence of proton, which makes it sensitive to the detection of changes in the signal; 4) diethylamino groups, which is known for their reversible response to protons. The designed probe Mito‐EFT facilitated the dynamic monitoring of mitochondrial electron flow micro‐dynamic alterations within living cells. Image created with Microsoft PowerPoint. Created in BioRender. Shao, S. (2024) BioRender.com/m64a243.

To address these challenges, we propose a four‐pronged labeling strategy targeting IMM protons to visualize Mito‐EF indirectly. This strategy entails transmembrane delivery devoid of membrane component interference, IMM targeting design, proton‐sensitive response groups, and a reversible release mechanism (**Figure** **1**). Specifically, we designed a probe called Mito‐EFT, incorporating a coumarin‐pyridine fluorescent tracer. Mito‐EFT is encapsulated in exosome‐sized nanoparticles to facilitate complete transmembrane delivery and minimize fluorescent interference from the cell membrane (Figure 1). Once in the cytoplasm, the probe's triple‐strength positive nitrogen charge efficiently targets the IMM (Figure 1). Additionally, the probe employs a diethylamino group for reversible proton response, enabling dynamic monitoring of Mito‐EF at the IMM. Using Mito‐EFT, we visualized Mito‐EF at the individual mitochondrion level for the first time (Figure 1). Furthermore, we investigated changes in Mito‐EF activity during various mitochondrial dynamic processes. Critically, we elucidated that Mito‐EF is regulated by the Dynamin‐Related Protein 1 (DRP1) protein, suggesting that our probe can be a powerful tool for assessing mitochondrial quality, surpassing traditional mitochondrial morphology evaluation. We also proposed a drug discovery approach combining Mito‐EF with advanced imaging technology, aiming to support the more precise identification of mitochondrial‐targeted drugs. The developed Mito‐EFT is an ideal candidate for dynamically tracking mitochondrial electron flow within biological systems.

## 2 Results

### 2.1 Mito‐EF‐Specific Fluorescent Molecular Probe Concept and Design Components

Although Mito‐EF is chemically invisible, its activity in the mitochondrial electron transport chain closely correlates with the rate of proton transfer across the inner mitochondrial membrane (IMM).^[^ ^17^ ^]^ Therefore, monitoring proton changes within the IMM could reflect Mito‐EF activity.^[^ ^18^ ^]^ We developed a probe design for Mito‐EF tracking (Mito‐EFT) to accomplish this. This design incorporates 1) coumarin,^[^ ^19^ ^]^ known for its exceptional optical performance in live cell imaging; 2) positive charges^[^ ^20^ ^]^ to target the IMM, ensuring precise probe localization; 3) diethylamino groups,^[^ ^20^ ^]^ which exhibit a reversible response to protons; and 4) encapsulation within exosomes^[^ ^21^ ^]^ to enhance transmembrane delivery and minimize fluorescence interference from the plasma membrane (Figure 1). Encapsulation of the Mito‐EFT probe within exosomes enhances its transmembrane transport efficiency. Guided by positive charges and attracted by the mitochondrial membrane potential, the probe reaches and settles on the IMM. There, Mito‐EFT captures electron signal transduction reversibly through its diethylamino group's response to protons, clearly depicting Mito‐EF activity across the IMM (Figure 1). In essence, our Mito‐EFT design offers direct and reversible monitoring of proton/Mito‐EF signals within the IMM (Figure 1). Below, we present detailed characterization and validation results of Mito‐EFT, and demonstrate its utility in imaging Mito‐EFT in live cells.

**Figure 2:**
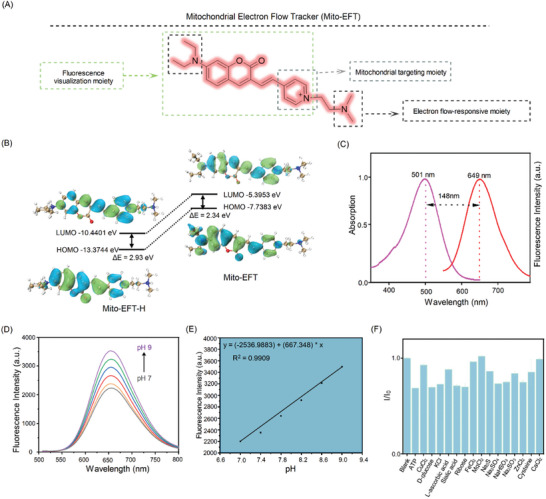
Design and the determination of physical and chemical properties of probe Mito‐EFT. A) Chemical structure of Mito‐EFT and the role of each functional moiety. The structure possesses a star for live cell imaging — coumarin, a positive charge that can target mitochondria and is known for its reversible response to protons — diethylamino. B) Calculated HOMO/LUMO distribution of Mito‐EFT‐H/Mito‐EFT based on the density functional theory. In both Mito‐EFT‐H and Mito‐EFT, the HOMO predominantly resided in the coumarin donor group, while the LUMO was primarily located in the pyridinium acceptor group. C) UV–vis absorption and the emission spectra of Mito‐EFT (10.0 µm) in UV/Vis absorption (purple solid lines) and emission (red solid lines) recorded in PBS (pH 7.4). Mito‐EFT revealed the maximum emission peak at 649 nm under 488 nm excitation. D) The fluorescence spectra of probe Mito‐EFT (10.0 µm) in the presence of various pH (7–9) in PBS, Ex, 488 nm. E) Correlation analysis of pH on the probe Mito‐EFT (10.0 µm) under different pH. Data are expressed as the mean ± SEM (n = 3). F) The fluorescent responses of Mito‐EFT (10.0 µm) in the presence of various biological analytes (100.0 µm) in PBS, Ex, 488 nm. Blank, ATP, CaCl_2_, CuCl_2_, D‐glucose, KCl, L‐ascorbic acid, sialic acid, ribose, FeCl_2_, FeCl_3_, MgCl_2_, Na_2_S, Na_2_SO_4_, NaHSO_3_, Na_2_SO_3_, ZnCl_2_, cysteine, and CaCl_2_.

Furthermore, we synthesized Mito‐EFT (**Figure** **2**A; Figure S1, Supporting Information) and confirmed its structure using standard methods (Figures S2–S4, Supporting Information). Notably, when Mito‐EFT was exposed to a proton‐lacking environment, the quaternary ammonium form of Mito‐EFT (Mito‐EFT‐H) transformed into the tertiary amine form of Mito‐EFT, accompanied by a gradual enhancement in its fluorescence signal. In both Mito‐EFT‐H and Mito‐EFT, the HOMO predominantly resided in the coumarin donor group, while the LUMO was primarily located in the pyridinium acceptor group, with an energy level gap ranging from 2.93 to 2.34 eV (Figure 2B). The proton flow response of Mito‐EFT‐H was attributed to the deprotonation of the donor within Mito‐EFT‐H under proton‐lacking conditions, facilitating an increased electron‐donating donor capacity and promoting Intramolecular Charge Transfer processes.^[^ ^22^ ^]^ Subsequently, we examined the fluorescence characteristics of Mito‐EFT in a phosphate‐buffered saline (PBS) solution (pH 7.4, 0.1 m), observing a Stokes shift of 148 nm for Mito‐EFT (Figure 2C), effectively mitigating potential interference from intracellular background signals. Given this, we investigated the response of Mito‐EFT to varying proton conditions by altering pH levels in PBS (Figures 2D,E; Figure S5, Supporting Information). After comprehensive testing, we confirmed that the Mito‐EFT recognition capacity remains consistently negative even under interference from various substances (Figure 2F).

Collectively, we have successfully developed the first Mito‐EFT probe, serving as a reliable indicator for depicting the dynamic changes in Mito‐EF.

### 2.2 Mito‐EF was Labeled by Mito‐EFT Probe in Living Cells

Initially, we evaluated the cytotoxicity of Mito‐EFT on HeLa cells and observed negligible toxic effects on cell activity (Figure S6, Supporting Information). The cell imaging experiment of co‐culturing Mito‐EFT with Hela cells shows that the optimal conditions are achieved with a Mito‐EFT concentration of 1 µm and an incubation time of 0.5 h (Figure S6, Supporting Information). Leveraging the nanoscale size of exosomes,^[^ ^23^ ^]^ we further confirmed this effect using super‐resolution microscopy (structured illumination microscopy, SIM), Mito‐EFT probes aggregated prominently at the cell membrane, whereas exosome encapsulation reversed this distribution (Figure S7, Supporting Information). Furthermore, upon entering cells, Mito‐EFT utilizes its ability to target positive charges in the mitochondrial cristae region, demonstrating significant colonization with the mitochondrial cristae‐specific dye, mito‐tracker (pKMTDR) (Figure S8, Supporting Information). Notably, the red particles labeled by Mito‐EFT exhibited an uneven distribution distinct from that of the pKMTDR labeling (Figure S8, Supporting Information). This localization pattern remained consistent across different cell lines, including HepG2 cells (Figure S9, Supporting Information), Human Skeletal Myoblasts (HSkM) cells (Figure S10, Supporting Information), and senescent HSkM cells (Figure S11, Supporting Information). Moreover, no colocalization with Mito‐EFT was observed with other intracellular membrane‐bound organelles, such as single‐layer membrane organelle‐lipid droplets (Figure S12, Supporting Information) and double‐layer membrane organelle‐lysosome (Figure S13, Supporting Information). These results confirm Mito‐EFT's ability to accurately locate the IMM area to respond to the target test object.

Additionally, the reversible binding ability of Mito‐EFT to Mito‐EF was confirmed by seahorse assay, revealing that the presence of different concentrations of Mito‐EFT did not interfere with mitochondrial baseline parameters with different incubating times, yielding negative results (Figure S14, Supporting Information). This finding validates the high reversibility of Mito‐EFT for binding Mito‐EF without interfering with mitochondrial biological function. Overall, our successful utilization of exosome‐mediated transmembrane delivery to target Mito‐EF within IMM and achieve reversible responses represents a visualization tool enabling the tracking of Mito‐EF's micro dynamics within mitochondria.

### 2.3 Mito‐EF Distribution Trend‐Dependent Mitochondrial Morphology Reported by Mito‐EFT

**Figure 3:**
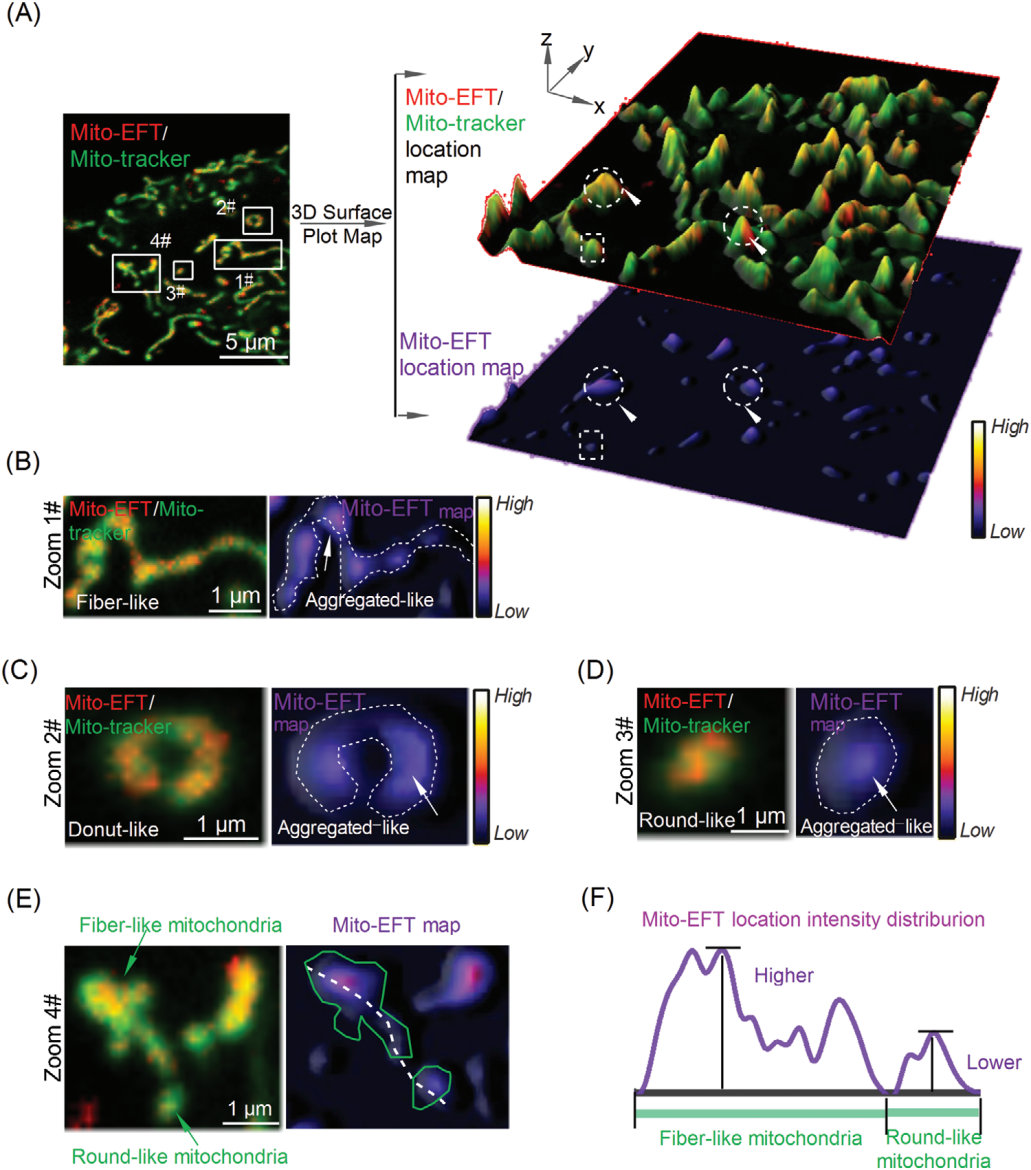
Mito‐EF distribution trend‐dependent mitochondrial morphology reported by Mito‐EFT. A) HeLa cells were stained with Mito‐EFT (1.0 µm) and Mito‐tracker (100.0 nm) for 30 min, and the 3D surface fluorescence distribution map of HeLa cells (Scale bar, 5 µm). B) Zoomed‐in images were of white rectangle #1 (A) that Mito‐EFT and Mito‐tracker tracking of fiber‐like mitochondria (Scale bar, 1 µm). C) Zoomed‐in images were of white rectangle #2 (A) that Mito‐EFT and Mito‐tracker tracking of donut‐like mitochondria (Scale bar, 1 µm). D) Zoomed‐in images were of white rectangle #3 (A) that Mito‐EFT and Mito‐tracker tracking of round‐like mitochondria (Scale bar, 1 µm). E) Mito‐EFT and Mito‐tracker tracking of fiber‐like and round‐like mitochondria simultaneously (Scale bar, 1 µm). F) Quantitative analysis of the Mito‐EFT location intensity distribution in (E). Mito‐EFT channel: Ex, 488 nm, Em, 600–650 nm; Mito‐tracker channel: Ex, 640 nm; Em, 641–694 nm.

Motivated by the observation that Mito‐EFT displayed a discontinuous distribution within round‐like mitochondria, exhibiting low Mito‐EFT signals, while fiber‐like mitochondria showed high signals (Figures S15, S16, Supporting Information), we further investigated the relationship between mitochondrial morphology and Mito‐EFT signals. We hypothesized that the heterogeneous distribution of Mito‐EF might correlate with mitochondrial shapes. To validate this hypothesis, we analyzed by double‐staining cells with Mito‐EFT and a commercial mitochondria tracker‐pKMTDR (**Figure** **3**A). Notably, pKMTDR labeled uniform mitochondrial 3D surface images. In contrast, the distribution of Mito‐EFT labeled fluorescence appeared irregular, displaying an aggregated‐like cluster pattern on fiber‐like, donut‐like, and round‐like mitochondria (Figures 3B–D; Figures S17–S19, Supporting Information), with a higher fluorescence signal within fiber‐like healthy mitochondria (Figure 3B; Figure S20, Supporting Information). Conversely, donut‐like and round‐like damaged mitochondria exhibited weaker Mito‐EFT fluorescence distribution (Figures 3C, D, Figures S21, S22, Supporting Information), indicating low Mito‐EF activity in damaged mitochondria and high Mito‐EF activity in healthy mitochondria (Figures 3E, F). In summary, these findings indicate differences in Mito‐EF between different mitochondrial morphology subpopulations, with a higher aggregation of Mito‐EF in fibrous‐like healthy mitochondria compared to round‐like damaged mitochondria.

### 2.4 Mito‐EF Tends to be Located at Mitochondrial Fission and Fusion Sites

**Figure 4:**
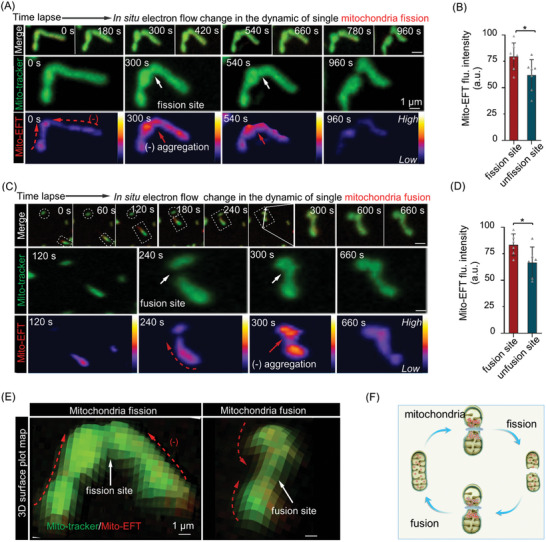
Mito‐EF tends to locate mitochondrial fission and fusion sites. A) The dynamic process of mitochondrial fission in HeLa cells stained with Mito‐EFT. Red arrows indicate Mito‐EF aggregation in the representative mitochondrial fission event. The time interval between each frame was ≈60 s (Scale bar, 1 µm). B) Quantitative analysis of Mito‐EFT (1.0 µm) at the fission sites in panel (A) (n = 7 images). Data are expressed as the mean ± SEM (n = 7, ^*^P < *0.05*). C) The dynamic process of mitochondrial fusion in HeLa cells stained with Mito‐EFT. Red arrows indicate Mito‐EF aggregation in the representative mitochondrial fusion event. The time interval between each frame was ≈60 s (Scale bar, 1 µm). D) Quantitative analysis of Mito‐EFT (1.0 µm) at the fusion sites in panel (C) (n = 6 images). Data are expressed as the mean ± SEM (n = 6, ^*^P < *0.05*). E) 3D distribution map of Mito‐EFT (1.0 µm) in the processes of mitochondrial fission and fusion (Scale bar, 1 µm). White arrows indicate the fission and fusion sites in the representative mitochondrial fission and fusion events. F) Schematic diagram of mitochondrial dynamic fission and fusion. Mito‐EFT channel: Ex, 488 nm, Em, 600–650 nm; Mito‐tracker channel: Ex, 647 nm; Em, 641–694 nm.

It is widely acknowledged that different subpopulations of mitochondrial morphologies result from dynamic mitochondria fission and fusion processes.^[^ ^24^ ^]^ To explore Mito‐EF's distribution during mitochondrial dynamics, we recorded mitochondrial fission and fusion events over 960 s (**Figure** **4**A). Notably, as a single mitochondrion labeled with Mito‐tracker into two distinct mitochondrial entities (Figure 4A, enlarged view, green color), Mito‐EF exhibited diverse localization patterns characterized by prominent expression at the fission site (Figure 4A, enlarged view, Mito‐EFT label, 0 s, red arrow), compared to non‐fissile mitochondria (Figure 4B). As fission progressed, Mito‐EF shifted from non‐fissile regions to the fission site, displaying intense fluorescence accumulation (Figure 4A, enlarged view, 300, 540 s). Subsequently, as fission concluded, Mito‐EF activity declined (Figure 4A, enlarged view, 960 s). We confirmed that photobleaching did not affect our observations, as continuous laser exposure for 960 s did not bleach the Mito‐EFT signal (Figure S23, Supporting Information). Figure S24 (Supporting Information) provides additional instances of Mito‐EF accumulation at fission sites, indicating its high motility characteristics in these regions. To corroborate these observations, we employed Mito‐EFT to track the Mito‐EF dynamic during mitochondrial fusion (Figure 4C). As anticipated, heightened Mito‐EF activity was observed at fusion sites, resulting in increased fluorescence (Figure 4D). These results further validate Mito‐EF's directional migration toward fission and fusion sites, actively contributing to mitochondrial morphological remodeling (Figures 4E, F ).

In summary, these findings support the concentration of Mito‐EF in regions of high activity, particularly at mitochondrial fission and fusion sites, where it plays an active role in mitochondrial quality control.

### 2.5 Drp1 Protein Regulates the Number of Mitochondrial Cristae for Modulating Mito‐EF Activity

**Figure 5:**
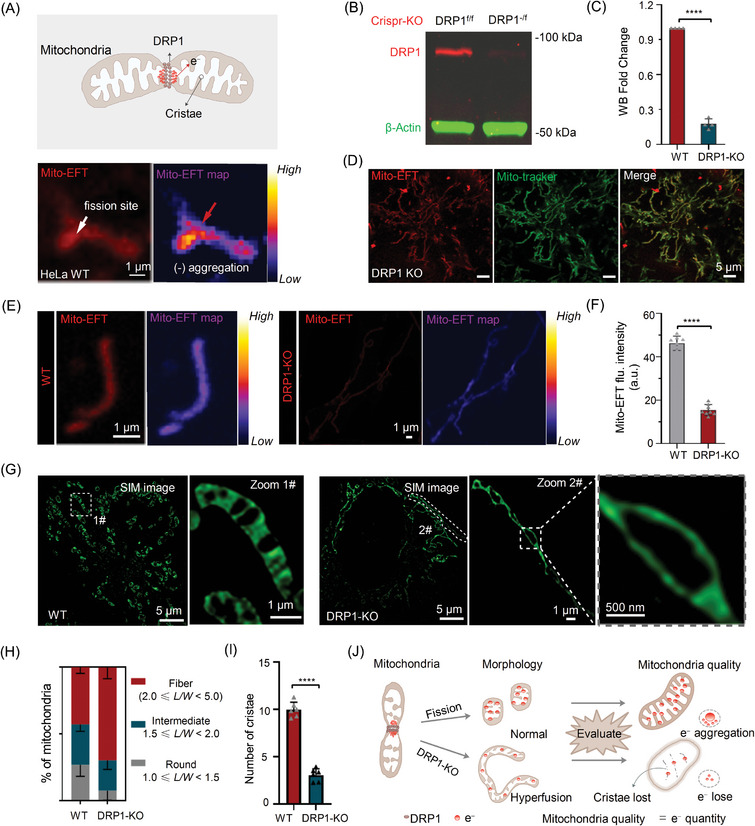
Drp1 regulates the number of mitochondrial cristae for modulating the Mito‐EF activity. A) The schematic diagram indicated that electrons were concentrated at the DRP1 concentration site during the mitochondrial fission event. The mitochondrial fission in HeLa cells stained with Mito‐EFT (Scale bar, 1 µm). 3D surface fluorescence distribution map perfectly shows the location of fluorescence. B) The expression of DRP1 after treatment with CRISPR/Cas9‐mediated DRP1 knockout in HeLa cells. C) Relative quantification results of western blotting. Data are expressed as the mean ± SEM (n = 3, ^****^P < *0.0001*). D) DRP1‐KO HeLa cells co‐stained with Mito‐tracker and Mito‐EFT (1.0 µm) for 30 min at 37 °C (Scale bar, 5 µm). E) The mitochondria in HeLa cells and DRP1‐KO HeLa cells were stained with Mito‐EFT (Scale bar, 1 µm). 3D surface fluorescence distribution map perfectly shows the location of fluorescence. F) Quantitative analysis of Mito‐EFT (1.0 µm) in different cells (n = 8 images). Data are expressed as the mean ± SEM (n = 8, ^****^P < *0.0001*). G) HeLa cells and DRP1‐KO HeLa cells co‐stained with mitochondrial membrane‐tracker (Scale bar, 5 µm). Zoomed‐in images were of white rectangles #1 and #2 indicating the mitochondrial cristae (Scale bar, 1 µm); the gray dotted line in zoomed‐in image #2 indicates the region for the loss of mitochondrial cristae (Scale bar, 500 nm). H) Mitochondrial morphology of the distribution parameters of *L/W*. I) Quantitative analysis of the number of mitochondrial cristae in different cells (n = 6 images). Data are expressed as the mean ± SEM (n = 6, ^****^P < *0.0001*). J) Schematic diagram of the mitochondrial quality evaluation process. During normal fission, mitochondria split into two, with normal cristae distribution and electron flow distribution. When DRP1 was knocked out, the mitochondria stopped fission and exhibited a hyperfusion state, and the cristae and electrons showed the same lost state. Therefore, mitochondrial quality is synchronized with electron quantity. Created in BioRender. Shao, S. (2024) BioRender.com/m64a243. Mito‐EFT channel: Ex, 488 nm, Em, 600–650 nm; and Mito‐membrane‐tracker channel: Ex, 647 nm; Em, 641–694 nm.

It is widely known that key proteins involved in mitochondrial quality control, such as DRP1^[^ ^25^ ^]^ are located at mitochondria fission sites, significantly influencing mitochondrial homeostasis (**Figure** **5**A). To further investigate the involvement of DRP1 protein in Mito‐EF relocalization during mitochondrial remodeling, we employed CRISPR/Cas9 to knockout (KO) the endogenous mitochondrial fission key protein DRP1 in HeLa cells (Figures 5B, C). Upon genetic inhibition of mitochondrial fission, the mitochondria displayed a hyperfused, fibrous morphology (Figure 5D), consistent with earlier findings.^[^ ^26^ ^]^ However, we noted a significant reduction in the fluorescent signal labeled by Mito‐EFT in DRP1‐KO cells, with some mitochondrial regions barely exhibiting detectable Mito‐EFT signal compared to wild‐type (WT) cells (Figures 5E, F). This outcome implies that DRP1‐KO alters mitochondrial morphology, thereby impeding Mito‐EF activity.

Numerous studies have underscored the involvement of Mito‐EF in the mitochondrial cristae region.^[^ ^27^ ^]^ To delve deeper into how the DRP1 protein regulates Mito‐EF activity, we hypothesized that DRP1 absence might disrupt mitochondrial cristae. Using super‐resolution microscopy to visualize mitochondrial cristae structures, we observed that while DRP1‐KO mitochondria showed a shift toward hyperfused mitochondrial subpopulation distribution via conventional mitochondrial morphology evaluation tools (Figure 5H), their cristae structures were nearly absent (Figure 5G, DRP1‐KO, enlarged view; Figure 5I). Traditional strategies for observing mitochondrial morphology fall short of capturing subtle changes in living cells.^[^ ^28^ ^]^ This finding highlights the limitation of conventional mitochondrial morphology assessments in characterizing mitochondrial quality and reflecting cristae structure (Figure 5J).

These findings support the association between the Mito‐EF signal and mitochondrial cristae maintained by DRP1 protein, underscoring the potential of our probe as a potent tool for assessing mitochondrial quality, surpassing traditional mitochondrial morphology evaluation methods.

### 2.6 Mito‐EF‐Driven Fluorescent Imaging Approach for Drug Discovery

**Figure 6:**
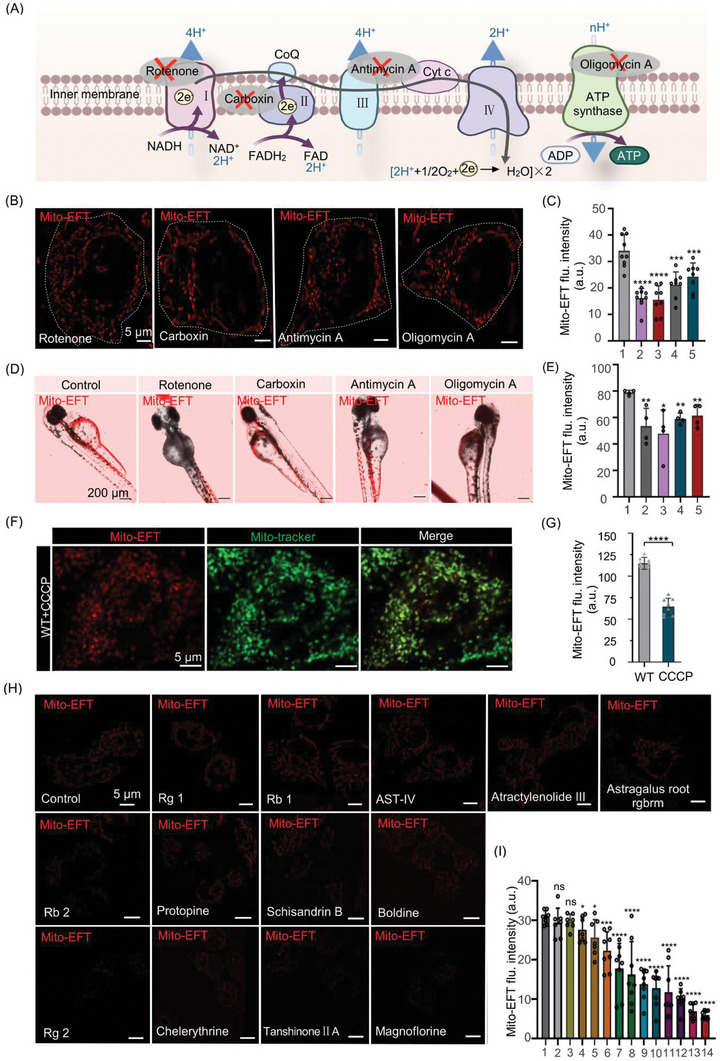
Mito‐EF‐driven fluorescent imaging approach for drug discovery. A) Schematic representation of oxidative phosphorylation inhibitors acting on the mitochondrial electron transport chain. Image created with Microsoft PowerPoint. B) Representative SIM images of HeLa cells incubated with Mito‐EFT (1.0 µm) for 30 min at 37 °C, followed by treatment with OXPHOS inhibitors (Scale bar, 5 µm). C) Quantitative analysis of the Mito‐EFT fluorescent intensity distribution in (B) (n = 8 images). 1. Untreated; 2. Rotenone; 3. Carboxin; 4. Antimycin A; and 5. Oligomycin A. Data are expressed as the mean ± SEM (n = 8, ^***^P < 0.001, ^****^P < *0.0001*). D) Representative SIM images of zebrafish incubated with Mito‐EFT at 37 °C, followed by treatment with OXPHOS inhibitors (Scale bar, 200 µm). E) Quantitative analysis of the Mito‐EFT fluorescent intensity distribution in (D) (n = 4 images). 1. Untreated; 2. Rotenone; 3. Carboxin; 4. Antimycin A; and 5. Oligomycin A. Data are expressed as the mean ± SEM (n = 4, ^*^P < 0.05, ^**^P < *0.01*). F) HeLa cells co‐stained with Mito‐tracker and Mito‐EFT (1.0 µm) for 30 min at 37 °C, followed by treatment with mitochondrial decoupling agent CCCP (Scale bar, 5 µm). G) Quantitative analysis of Mito‐EFT (1.0 µm) under different treatments (n = 8 images). Data are expressed as the mean ± SEM (n = 8, ^****^P < *0.0001*). H) Representative SIM images of HeLa cells incubated with Mito‐EFT (1.0 µm) for 30 min at 37 °C, followed by treatment with different active substances in Traditional Chinese Medicine (Scale bar, 5 µm). I) Quantitative analysis of the Mito‐EFT fluorescent intensity distribution in (H) (n = 7 images). 1. Untreated; 2. Panaxoside Rg1; 3. Ginsenoside Rb1; 4. Astragaloside IV; 5. Atractylenolide III; 6. Astragalus Root Rgbrm; 7. Ginsenoside Rb2; 8. Protopine; 9. Schisandrin B; 10. Boldine; 11. Ginsenoside Rg2; 12. Chelerythrine; 13. Tanshinone IIA; and 14. Magnoflorine. Data are expressed as the mean ± SEM (n = 7, n.s., ^*^P < 0.05, ^**^P < 0.01, ^***^P < 0.001, ^****^P < *0.0001*). Mito‐EFT channel: Ex, 488 nm, Em, 600–650 nm; Mito‐tracker channel: Ex, 640 nm; and Em, 641–694 nm.

Recognizing that mitochondrial morphological parameters alone do not reflect true mitochondrial inner vitality for drug progress assessment, we introduce a Mito‐EF‐driven fluorescent imaging drug screening approach. To validate it, we tested Mito‐EFT's sensitivity to Mito‐EF using representative inhibitors of oxidative phosphorylation complexes I, II, III, and ATP synthase, such as Rotenone, carboxin, antimycin A, and Oligomycin (**Figure** **6**A). Results showed that Mito‐EFT exhibited fluorescent inhibition signals in response to these inhibitors, with the fluorescence changes correlating well with the sequence of electron transfer chain inhibition (Figures 6B, C). Moreover, mitochondrial morphologies (fibrous, donut‐shaped, round) treated with these inhibitors displayed corresponding decreases in fluorescent signals (Figures S25–S27, Supporting Information). Zebrafish imaging experiments were also confirmed with cell imaging results^[^ ^29^ ^]^ (Figures 6D, E). Additionally, we explored the relationship between mitochondrial EF and mitochondrial quality by using the mitochondrial uncoupler carbonyl cyanide m‐chlorophenyl hydrazone (CCCP) as a positive control. The results indicated that CCCP treatment reduced Mito‐EF activity and increased round mitochondria (Figures 6F, G), supporting the reliability of Mito‐EFT in detecting Mito‐EF and assessing mitochondrial quality.

Subsequently, we screened 13 small‐molecule drugs using this approach (Figures 6H, I).^[^ ^30a–d^ ^]^ Among them, Chererythrine, Tanshinone IIA, and Magnoflorine significantly reduced mitochondrial electron flow. Notably, Magnoflorine exhibited the most profound effect on Mito‐EFT, indicating its potential as a mitochondrial‐targeted drug. To validate this, leveraging Magnoflorine's autofluorescent properties, we further investigated its subcellular localization, revealing a high co‐localization with mitochondria (Figure S28, Supporting Information). This finding further substantiates our Mito‐EFT drug screening method's precision in identifying drugs that directly target mitochondria.

**Figure 7:**
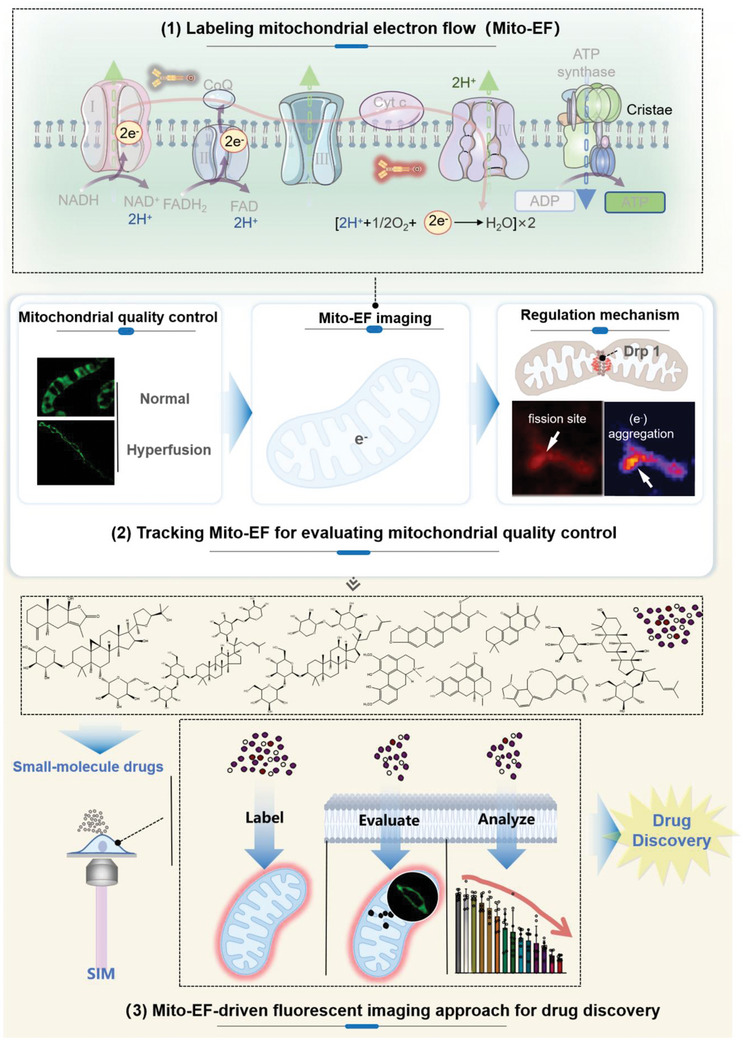
Schematic diagram of Mito‐EF‐driven fluorescent imaging approach for drug discovery. First, the mitochondrial electron flow tracker, Mito‐EFT, provides comprehensive labeling of mitochondrial electron flow (Mito‐EF). In this study, we developed Mito‐EFT, a novel molecular probe specifically designed to track and visualize Mito‐EF. This probe allows for the evaluation of different mitochondrial morphologies through mitochondrial electron flow analysis. Mito‐EF tends to aggregate in fibrous, healthy mitochondria and shows lower aggregation in round, damaged mitochondria, highlighting its potential role in maintaining mitochondrial homeostasis. Additionally, the significant association between fission sites controlled by DRP1 protein and Mito‐EF underscores the complex interactions between mitochondrial structure and function. Our observations also indicated the localization trend of Mito‐EF at mitochondrial fission and fusion sites, suggesting its active involvement in mitochondrial dynamics and remodeling processes. The directional migration of Mito‐EF emphasizes its crucial role in mitochondrial quality control and the necessity of its regulatory mechanisms. By monitoring Mito‐EFT in drug induction experiments and animal models, we confirmed its reliability in responding to Mito‐EF activity under various conditions. Furthermore, Mito‐EFT facilitates a fluorescence imaging approach for assessing the impact of active ingredients in traditional Chinese Medicine on mitochondrial electron flow. This method integrates newly developed electron flow tracers and advanced imaging techniques, allowing for accurate evaluation of mitochondrial activity without relying on traditional morphology‐based drug discovery strategies. This enables precise evaluation and analysis of drugs, providing a powerful tool for drug discovery. Image created with Microsoft PowerPoint. Created in BioRender. Shao, S. (2024) BioRender.com/m64a243.

In summary, we introduce a novel drug‐screening approach driven by subcellular fluorescence imaging (**Figure** **7**). This method integrates newly developed electron flow tracers and advanced imaging techniques, enabling precise insights into mitochondrial activities independent of traditional mitochondrial morphology‐based drug discovery strategies.

## 3 Conclusion

In this study, we successfully developed a novel molecular probe, Mito‐EFT, tailored for tracking and visualizing Mito‐EF. Demonstrating its efficacy in live cell monitoring, we unveiled its pivotal role in modulating mitochondrial quality control mechanisms. Notably, Mito‐EF exhibited a tendency to aggregate in fibrous‐like healthy mitochondria, contrasting with its lower aggregation in round‐like damaged mitochondria, suggesting a correlation between Mito‐EF distribution and mitochondrial morphology, highlighting the potential role of Mito‐EF in maintaining mitochondrial homeostasis. Furthermore, our findings underscored the dynamic relocation of Mito‐EF at mitochondrial fission and fusion sites, implicating its active involvement in mitochondrial morphological remodeling.

The significant association between Mito‐EF and mitochondrial cristae, controlled by the DRP1 protein, highlights the intricate interplay between mitochondrial structure and function.^[^ ^31^ ^]^ Utilizing the Mito‐EFT probe, we visualized and tracked Mito‐EF activity within individual mitochondria, offering insights beyond traditional morphology evaluation methods. Moreover, our observations revealed Mito‐EF's tendency for localization at mitochondrial fission and fusion sites, indicating its active involvement in mitochondrial dynamics and reshaping processes. This directional migration of Mito‐EF emphasizes its crucial role in mitochondrial quality control, highlighting the necessity of investigating its regulatory mechanisms for overall mitochondrial health. In addition, Mito‐EFT has demonstrated reliable monitoring capabilities in drug induction experiments and animal models. Additionally, our results affirm the versatility and reliability of Mito‐EFT in responding to Mito‐EF activity under diverse conditions, underscoring its potential as a valuable tool for mitochondrial biology research. However, its emission wavelength of 488 nm precludes in vivo experiments and hampers clinical trial application, necessitating further structural optimization.

In conclusion, our study introduces a new approach to understanding how Mito‐EF regulates mitochondrial quality control. Using a developed Mito‐EF tracker and advanced microscopy, we revealed the dynamic mechanisms behind Mito‐EF's role in maintaining mitochondrial homeostasis. These findings enhance our understanding of Mito‐EF dynamics and open avenues for exploring complex mechanisms governing mitochondrial function. Our study also suggests that Mito‐EFT can be a reliable indicator for assessing mitochondrial quality, outperforming traditional methods. Overall, our research provides valuable insights into the critical role of Mito‐EF in mitochondrial health, offering opportunities for further exploration and drug discovery in cellular health and disease.

## 4 Experimental Section

### Materials and Measurements

The reagents and solvents were procured from Macklin or Aladdin companies. Fetal bovine serum (FBS) was sourced from VivaCell Shanghai (Shanghai, China), while Dulbecco's modified Eagle's medium (DMEM), Penicillin‐streptomycin (10000 units mL^−1^), Trypsin‐EDTA phenol‐free medium (obtained from Gibco, Grand Island, NY, USA), and other essential reagents for cell culture were acquired from Gibco BRL (Grand Island, NY, USA). Specific staining reagents, including pKMTDR (Mito‐Tracker Deep Red), and Lipi‐Blue, were obtained from Invitrogen (Eugene, Oregon, USA). Cell Counting Kit‐8（CCK‐8；CT0001‐B） were purchased from Shandong Sparkjade Biotechnology Co., Ltd. (Shandong, China). The HepG2 cells were generously provided by Fengshan Wang's laboratory at Shandong University, while HeLa and HSkM cells were graciously shared by Xiaodong Mu's laboratory at Shandong First Medical University. Zebrafish were graciously shared by Liwen Han's laboratory at Shandong First Medical University. The DRP1 knockout HeLa cells were obtained from Cyagen. RIPA lysis buffer was purchased from Millipore, whereas LDS sample buffer was procured from Invitrogen. Essential materials such as polyacrylamide gels, nitrocellulose membranes, and protein standards were sourced from Bio‐Rad. Antibodies were acquired from the Odyssey.

### Theoretical Calculations

For density functional theory calculations, the Gaussian16 program was employed at the B3LYP/6‐31G(d) level, considering both Mito‐EFT‐H and Mito‐EFT. Subsequent data analysis was carried out using Multiwfn and VMD 1.9.3 software tools.

### Cell Culture

HeLa cells were cultured in DMEM supplemented with 10% FBS, 100 µm of penicillin, and 100 µg mL^−1^ of streptomycin. The cells were maintained at 37 °C in a humidified atmosphere containing 5% CO_2_.

### Cytotoxicity Assay

Cytotoxicity assessment was conducted using the CCK‐8 assay. HeLa cells were seeded into 96‐well plates at a density of 8 × 10^3^ cells per well, and cultured in DMEM with 10% FBS for 36 h in an incubator maintained at 5% CO_2_ and 37 °C. Subsequently, the culture medium was replaced with 100 µL of fresh medium containing varying concentrations (0, 5.0, 10.0, 20.0, and 30.0 µm) of Mito‐EFT. After 24 h of incubation, a mixture containing 10% CCK‐8 solution was added to each well, followed by a further 2 h of incubation under the same conditions. The absorbance of each well was then measured at 450 nm using a fluorescence microplate reader.

(1) $$\begin{array}{ccc} \mathrm{Cell}\;\mathrm{viability} & = & [(\mathrm{experimental}\;\mathrm{holes}-\mathrm{empty}\;\mathrm{holes})/\\ & & \left(\mathrm{contrast}\;\mathrm{holes}-\mathrm{empty}\;\mathrm{holes})\right] \end{array}$$

### Oxygen Consumption Rate (OCR) Measurement

HeLa cells, both untreated and those exposed to Mito‐EFT, were plated at a density of 1.0 × 10^4^ cells per well in XFe96 cell culture plates (Agilent Technologies, USA). Following 24 h of incubation, the culture medium (DMEM) was aspirated, and it was replaced with XF DMEM Medium, pre‐warmed and supplemented with 1 mm sodium pyruvate, 10.0 mm glucose, and 2 mm L‐glutamine, with the pH set at 7.4. Subsequently, all cells were treated with specific compounds, including 1 µm oligomycin A, 1 µm FCCP, and 500 nm rotenone/antimycin A. The assessment of oxygen consumption rates (OCRs) in the cells was conducted using the XF Cell Mito Stress Test Kit (Agilent Technologies, USA), and the Seahorse XF96 analyzer (Agilent Technologies, USA) was utilized for the measurement of OCR.

### Structure Illumination Microscopy and Confocal Laser Scanning Microscopy

The images were obtained using Elyra 7 and the LSM‐980 confocal laser scanning microscope (Carl Zeiss, Inc.) equipped with a 63 × /1.49 numerical aperture oil‐immersion objective lens and were analyzed with ZEN 2012 (Carl Zeiss, Inc.) and ImageJ software (National Institutes of Health).

### Colocalization Experiments

HeLa cells were initially plated at a density of 1 × 10^5^ cells on 35 mm glass‐bottom culture dishes and incubated with 2 mL of DMEM containing 10% FBS for 24 h (maintained at 5% CO_2_ and 37 °C). Following this, HeLa cells were treated with 1.0 µm of Mito‐EFT for 30 min, and 100 nm of Mito‐Tracker Deep Red (pKMTDR) for 30 min. Subsequently, the cells were subjected to three washes with PBS, followed by two washes with fresh DMEM. Finally, the cells were cultured in 1 mL of phenol‐free medium and subjected to imaging using confocal laser scanning microscopy (CLSM). pKMTDR was excited at 647 nm and emitted within the range of 641–694 nm. The acquired images were subsequently processed and analyzed using ImageJ software.

### Exosomes Separation Experiments

Exosomes derived from hUCMSCs (Exo^hUCMSCs^) were isolated by differential centrifugation. To harvest exosomes, 200 mL cell culture media were collected. Cell debris was removed from the cell culture supernatant by centrifugation at 300 and 2000 g, and each rotational speed was for 10 min. To remove non‐exosome vesicles and any possible apoptotic bodies, the supernatants were then spun at 10000 g for 40min. Finally, exosomes were obtained at 120000 g for 70 min. All centrifugations were done at 4 °C. Exosomes were used directly or stored at −80 °C for follow‐up experiments.

### Exosomes Package Mito‐EFT Experiment

Uptake of Exo^hUCMSCs^ was analyzed through co‐cultured HeLa with DIL‐labeled Exo^hUCMSCs^, as reported previously. Briefly, DIL‐labeled Exo^hUCMSCs^ were used and co‐incubated with the Mito‐EFT at 37 °C for 0.5 h. Subsequently, the HeLa was washed with sterile PBS to remove uninternalized Exo^hUCMSCs^, and the DIL‐labeled Exo^hUCMSCs^ (purple dots) were observed by microscope.

### OXPHOS Inhibitors Experiments

HeLa cells were initially plated at a density of 1 × 10^5^ cells on 35 mm glass‐bottom culture dishes and incubated with 2 mL of DMEM containing 10% FBS for 24 h (maintained at 5% CO_2_ and 37 °C). Following this, Cells were pre‐incubated with a combination of Rotenone (10.0 µm); Carboxin (10.0 µm); Antimycin A (10.0 µm), and Oligomycin A (10.0 µm) in FBS DMEM at 37 °C for 12 h, followed by incubation with Mito‐EFT at 37 °C for 30 min; After these treatments, the cells were imaged using structure illumination microscopy, and the resulting fluorescence intensities were subjected to quantitative analysis.

### CRISPR/Cas9 Mediated DRP1 Knockout

The deletion of HIF‐1α in HeLa cell lines was carried out by Cyagen (China). In a succinct description of the methodology, the gene sequence of DRP1 was sourced from the NCBI database, and sgRNA plasmids were designed and subsequently constructed. Employing the CRISPR/Cas9 gene editing technique, the Cas9 and sgRNA plasmids were introduced into HeLa cell lines through electroporation. Subsequently, monoclonal cells featuring CRISPR/Cas9‐mediated DRP1 deletions were screened and isolated with the aid of PCR. Finally, homozygote verification was accomplished through the application of Sanger sequencing.

### Fluorescent Western Blotting

WT‐HeLa and DRP1‐KO HeLa cells were initially subjected to two PBS washes on ice, followed by lysis in RIPA buffer that included additional protease and phosphatase inhibitors. The obtained lysates were maintained on ice for 20 min, then centrifuged for 15 min at 12000 rpm at 4 °C. The resultant supernatants were subjected to the BCA Protein Assay Kit to quantify total protein concentrations. The samples were combined with loading buffer and loaded onto 12.5% polyacrylamide gels for subsequent SDS‐PAGE, conducted at room temperature. After electrophoresis, the samples were transferred onto nitrocellulose membranes at a constant current of 200 mA, maintained at 4 °C for 1 h. These membranes were then blocked in a 5% BSA/TBST solution for 1.5 h at room temperature, and subsequently incubated with primary antibodies and fluorescent secondary antibodies in accordance with the manufacturer's recommendations. Membranes were visualized using the Odyssey Dix imaging system. Signal analysis was carried out employing ImageJ software, and the activity was defined as the signal ratio of phospho‐target/total target.

### Zebrafish Uptake Test

7 dpf zebrafish embryos were rinsed with embryonic culture medium (E3 medium, 5 mm NaCl, 0.17 mm KCl, 0.33 mm CaCl_2_, 0.33 mm MgSO_4_, pH 7.4). Following this, zebrafish embryos were pre‐incubated with a combination of Rotenone (10.0 µm); Carboxin (10.0 µm); Antimycin A (10.0 µm), and Oligomycin A (10.0 µm) in FBS DMEM at 37 °C for 12 h, followed by incubation with Mito‐EFT at 37 °C for 30 min; After incubation, embryos were anesthetized with 0.02% tricaine for 1 min and the fluorescence images was detected by confocal microscope. The fluorescence intensity of zebrafish embryos was quantified by Image J software.

### Drug Screening Experiment

Cells were initially plated at a density of 1 × 10^5^ cells on 35 mm glass‐bottom culture dishes and incubated with 2 mL of DMEM containing 10% FBS for 24 h (maintained at 5% CO_2_ and 37 °C). Following this, Cells were pre‐incubated with a combination of Panaxoside Rg1 (10.0 µm); Ginsenoside Rb1 (10.0 µm); Astragaloside IV (10.0 µm); Atractylenolide III (10.0 µm); Astragalus Root Rgbrm (10.0 µm); Ginsenoside Rb2 (10.0 µm); Protopine (10.0 µm); Schisandrin B (10.0 µm); Boldine (10.0 µm); Ginsenoside Rg2 (10.0 µm); Chelerythrine (10.0 µm); Tanshinone IIA (10.0 µm) and Magnoflorine (10.0 µm). in FBS DMEM at 37 °C for 12 h, followed by incubation with Mito‐EFT at 37 °C for 30 min; After these treatments, the cells were imaged using structure illumination microscopy, and the resulting fluorescence intensities were subjected to quantitative analysis.

### Data Analysis

Statistical analysis was carried out using Origin 2018, GraphPad Prism 8, and ImageJ software. Normality and lognormality test was used to check the normal distribution. In the case of normal distribution, the statistical comparison of results was done with a Student's t‐test. In the case of non‐normal distribution, the statistical comparison of results was done with a Mann–Whitney test, with levels of significance set at n.s. (no significant difference), ^*^P < 0.05, ^**^P < 0.01, ^***^P < *0.001*, and ^****^P < *0.0001*. Data are presented as mean ± SEM. Analyzed cells were obtained from three replicates. Statistical significances and sample sizes in all graphs are indicated in the corresponding Figure legends.

## Conflict of Interest

The authors declare no conflict of interest.

## Author Contributions

Y.R., L.‐L.W., W.S., and Y.G. contributed equally to this work. Y.R. did investigation, formal analysis, and wrote the original draft. L.W., W.S., L.S., Y.G. did reviewing and editing. X.S., Z.L., Z.Y. did validation and wrote the original draft. S.W. M.Z. performed methodology and visualization. L.H. J.W. performed methodology and visualization. X.S., K.Z., Q. C. did reviewing and editing.

## Supporting information



Supporting Information

## Data Availability

The data that support the findings of this study are available from the corresponding author upon reasonable request.
