# Supplementary material for: Mitochondrial Electron Flow Dynamics Imaging for Assessing Mitochondrial Quality and Drug Screening
Source: Adv Sci (Weinh). 2025 Jan 13;12(9):2410561. doi: 10.1002/advs.202410561 (PMC11884618; doi:10.1002/advs.202410561)
Supplement: Supplementary file 1 — Supporting Information [file ADVS-12-2410561-s001.docx]

Supplemental information

Mitochondrial Electron Flow Dynamics Imaging for Assessing Mitochondrial Quality and Drug Screening

*Youxiao Ren^1,†^, Ling-Ling Wu^1,†^,* *Wenjing Song^1,†^,Yanan Gao^1,†^,Litao Shao^4^, Zhiyuan Lu^1^, Songsong Wang^1^, Xintian Shao^1*^, Zhenjie Yu**^1^, Mengrui Zhang^1^, Jing Wu^3^, Liwen Han^1^, Kewu Zeng^1,2*^, Qixin Chen^1^**^,5*^*

**Table of Contents**

**Figure S1.** Synthetic scheme of Mito-EFT.

**Figure S2.** ^1^H NMR spectra of Mito-EFT in DMSO**-***d*_6_.

**Figure S3.** ^13^C NMR spectra of Mito-EFT in DMSO-*d*_6_.

**Figure S4.** High-resolution mass spectrum of Mito-EFT.

**Figure S5.** Fluorescence spectra of probe Mito-EFT (10.0 μM) in the presence of various pH (2 - 11) in PBS (pH 7.4).

**Figure S6.** Cytotoxicity testing and cell imaging experiment of co-incubation of Mito-EFT with Hela cells.

**Figure S7.** Mito-EF was labeled by Mito-EFT probe in living cells.

**Figure S8.** Colocalization images of Mito-EFT (1.0 μM) with Mito-tracker in HeLa cells.

**Figure S9.** Colocalization images of Mito-EFT (1.0 μM) with the commercial mitochondrial probe in HepG2 cells.

**Figure S10.** Colocalization images of Mito-EFT (1.0 μM) with the commercial mitochondria probe in human skeletal muscle cells HSkM.

**Figure S11.** Colocalization images of Mito-EFT (1.0 μM) with the commercial mitochondria probe in senescent HSkM cells.

**Figure S12.** Colocalization images of Mito-EFT (1.0 μM) with lipid droplet in HeLa cells.

**Figure S13.** Figure S13. Colocalization images of Mito-EFT (1.0 μM) with lysosome in HeLa cells.

**Figure S14.** The mitochondrial respiratory function was examined using the OCR assay.

**Figure S15.** Mitochondrial distribution of different morphology.

**Figure S16.** Characterization of Mito-EFT in living cells.

**Figure S17.** Colocalization images of Mito-EFT (1.0 μM) with Mito-tracker in fiber-like mitochondria.

**Figure S18.** Colocalization images of Mito-EFT (1.0 μM) with Mito-tracker in donut-like mitochondria.

**Figure S19.** Colocalization images of Mito-EFT (1.0 μM) with Mito-tracker in round-like mitochondria.

**Figure S20.** Mito-EFT tracking of fiber-like mitochondrial morphology.

**Figure S21.** Mito-EFT tracking of donut-like mitochondrial morphology.

**Figure S22.** Mito-EFT tracking of round-like mitochondrial morphology.

**Figure S23.** Long-term imaging of the Mito-EFT.

**Figure S24.** Long-term imaging of the Mito-tracker and Mito-EFT.

**Figure S25.** The fluorescence intensity changes of Mito-EFT in the tracking of fiber-like mitochondria treated with OXPHOS inhibitors.

**Figure S26.** The fluorescence intensity changes of Mito-EFT in the tracking of donut-like mitochondria treated with OXPHOS inhibitors.

**Figure S27.** The fluorescence intensity changes of Mito-EFT in the tracking of round-like mitochondria treated with OXPHOS inhibitors.

**Figure S28.** Colocalization images of MF (10.0 μM) with the commercial mitochondrial probe in HeLa cells.


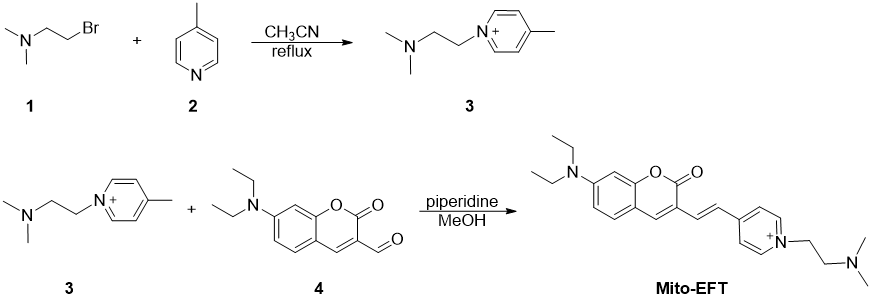


**Figure S1.** **Synthetic scheme of Mito-EFT.**

Compound **3**: Compound **1** (2-bromo-N, N-dimethylethan-1-amine, 228 mg, 1.5 mmol) and Compound **2** (4-methylpyridine, 236 mg, 1.0 mmol) were dissolved into acetonitrile (6 mL) at room temperature under Argon, and the reaction mixture was stirred at 80 °C under Argon for 12 h. After cooling to room temperature, the reaction mixture was added into cold ether (100 mL) dropwise. Compound **3** was obtained as a white powder by precipitation and filtration, and used directly without further purification.

Mito-EFT**:** To a solvent of compound **3** (33 mg, 0.2 mmol) and compound **4** (49 mg, 0.2 mmol) in absolute ethanol (6 mL), piperidine (73.4 mg, 0.8 mmol) was added at room temperature under Argon. The reaction mixture was stirred at room temperature under Argon for 12 h. and then added into cold ethyl acetate (100 mL) dropwise. After precipitation and filtration, Mito-EFT was obtained as a red powder, 51 mg, 65% yield. ^1^H NMR (400 MHz, DMSO-*d*_6_) *δ* 8.84 (d, *J* = 6.8 Hz, 2H), 8.27 (s, 1H), 8.18 (d, *J* = 6.9 Hz, 2H), 7.87 (d, *J* = 16.0 Hz, 1H), 7.69 (d, *J* = 16.0 Hz, 1H), 7.56 (d, *J* = 9.0 Hz, 1H), 6.81 (dd, *J* = 9.0, 2.3 Hz, 1H), 6.61 (d, *J* = 2.2 Hz, 1H), 4.65 – 4.51 (m, 2H), 3.49 (q, *J* = 7.0 Hz, 4H), 2.76 (d, *J* = 5.5 Hz, 2H), 2.21 (s, 6H), 1.15 (t, *J* = 7.0 Hz, 6H). ^13^C NMR (150 MHz, DMSO-*d*_6_) *δ* 160.06, 156.81, 153.80, 152.49, 145.79, 144.75, 137.38, 131.19, 123.36, 123.16, 114.24, 110.54, 108.89, 96.77, 59.05, 57.33, 45.44, 44.85, 12.86. HRMS: (ESI, m/z): [M]^+^ calcd. for C_24_H_30_N_3_O_2_^+^: 392.2333; found: 392.2343.


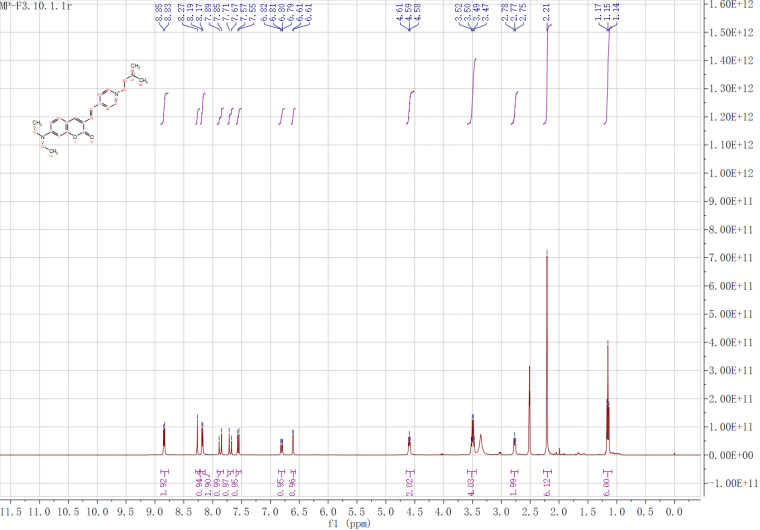


**Figure S2. ^1^H NMR spectra of Mito-EFT in DMSO-*d*_6_.**


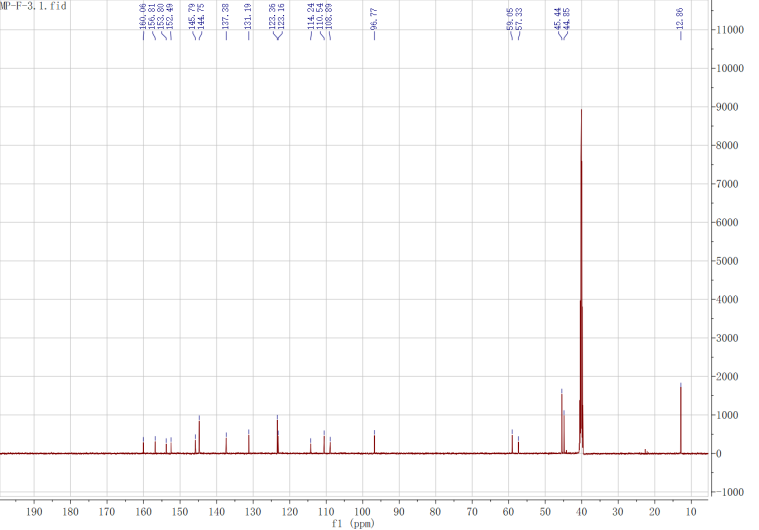


**Figure S3.** **^13^C NMR spectra of Mito-EFT in DMSO-*d*_6_.**


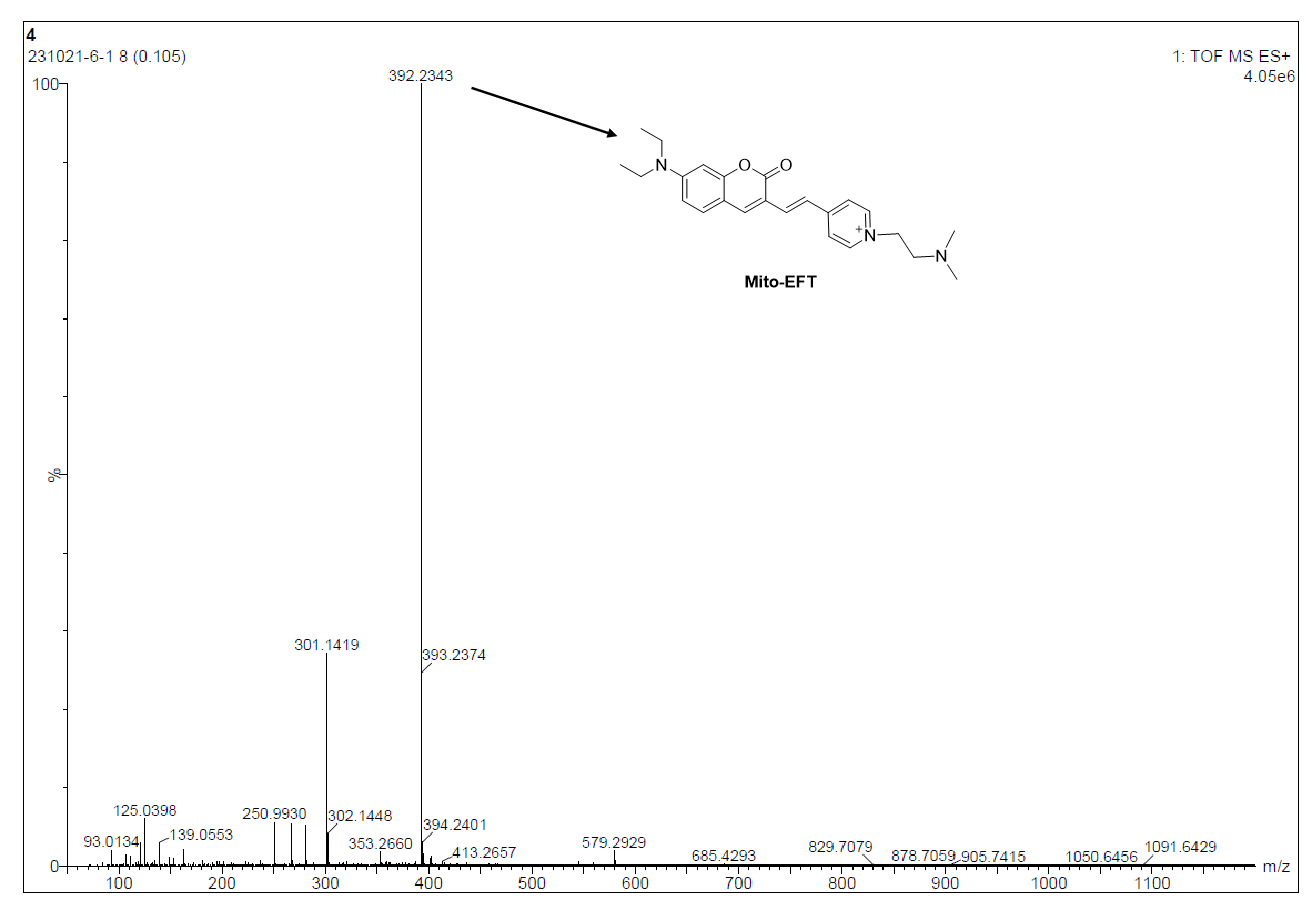


**Figure S4.** **High-resolution mass spectrum of** **Mito-EFT.**


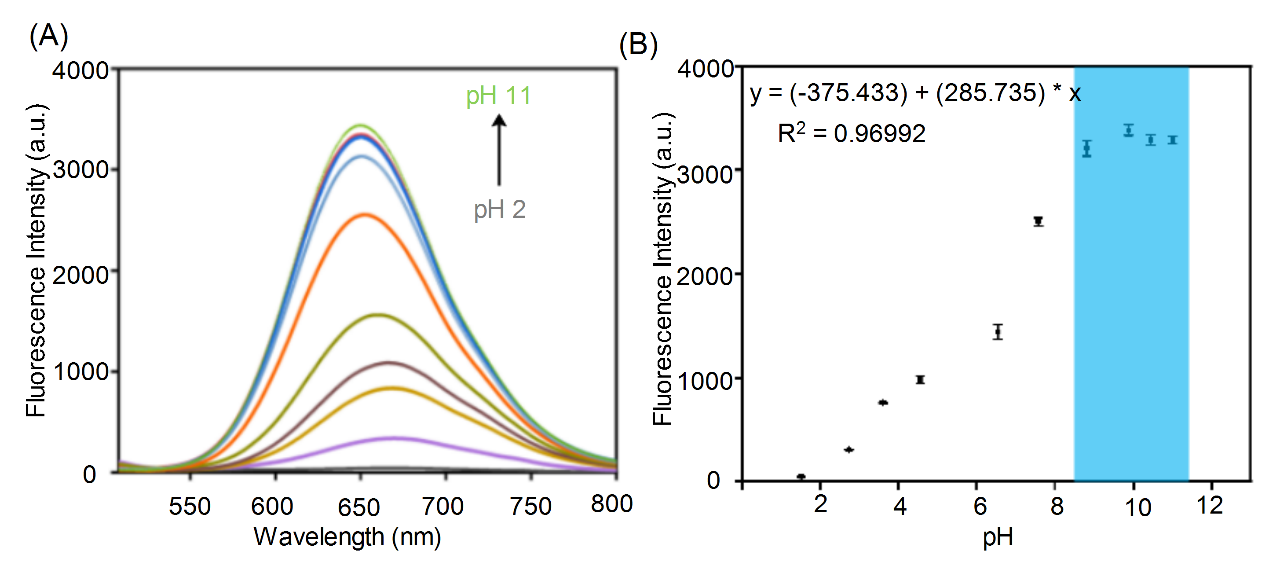


**Figure S5. Fluorescence spectra of probe Mito-EFT (10.0 μM) in the presence of various pH (2 - 11) in PBS.**

(A) Fluorescence spectra of probe Mito-EFT (10.0 μM) in the presence of various pH (2 - 11) in PBS, Ex, 488 nm.

(B) Effects of pH on the probe Mito-EFT (10.0 μM) under different pH. Data are expressed as the mean ± SEM (n = 3).


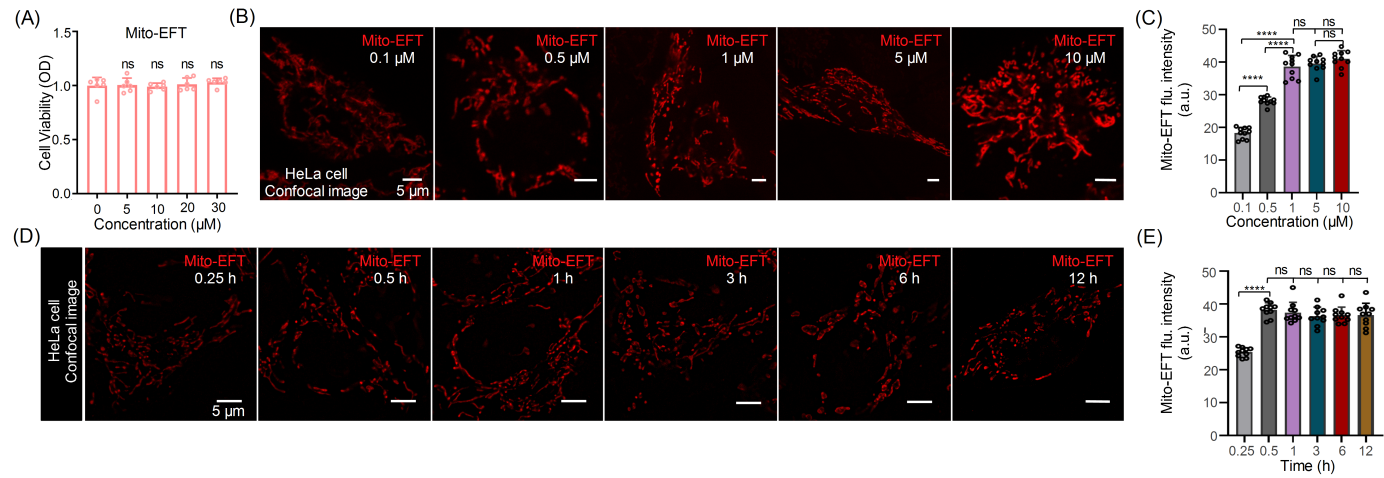


**Figure S6.** **Cytotoxicity testing and cell imaging experiment of co-incubation of Mito-EFT with Hela cells.**

1. Viability of HeLa cells treated with the various concentrations of Mito-EFT for 24 h.

(B) Fluorescence images of HeLa cells incubated with different concentrations of Mito-EFT (Scale bar, 5 μm).

(C) Quantitative analysis of the Mito-EFT fluorescent intensity distribution of HeLa cells treated with different concentrations of Mito-EFT. (n = 10 images)

(D) Fluorescence images of HeLa cells incubated with different times of Mito-EFT (Scale bar, 5 μm).

(E) Quantitative analysis of the Mito-EFT fluorescent intensity distribution of HeLa cells treated with different times of Mito-EFT. (n = 10 images)

Data were expressed as the mean ± SEM (n = 10, n.s., *****P < 0.0001*). Mito-EFT channel: Ex, 488 nm, Em, 600 - 650 nm.

**
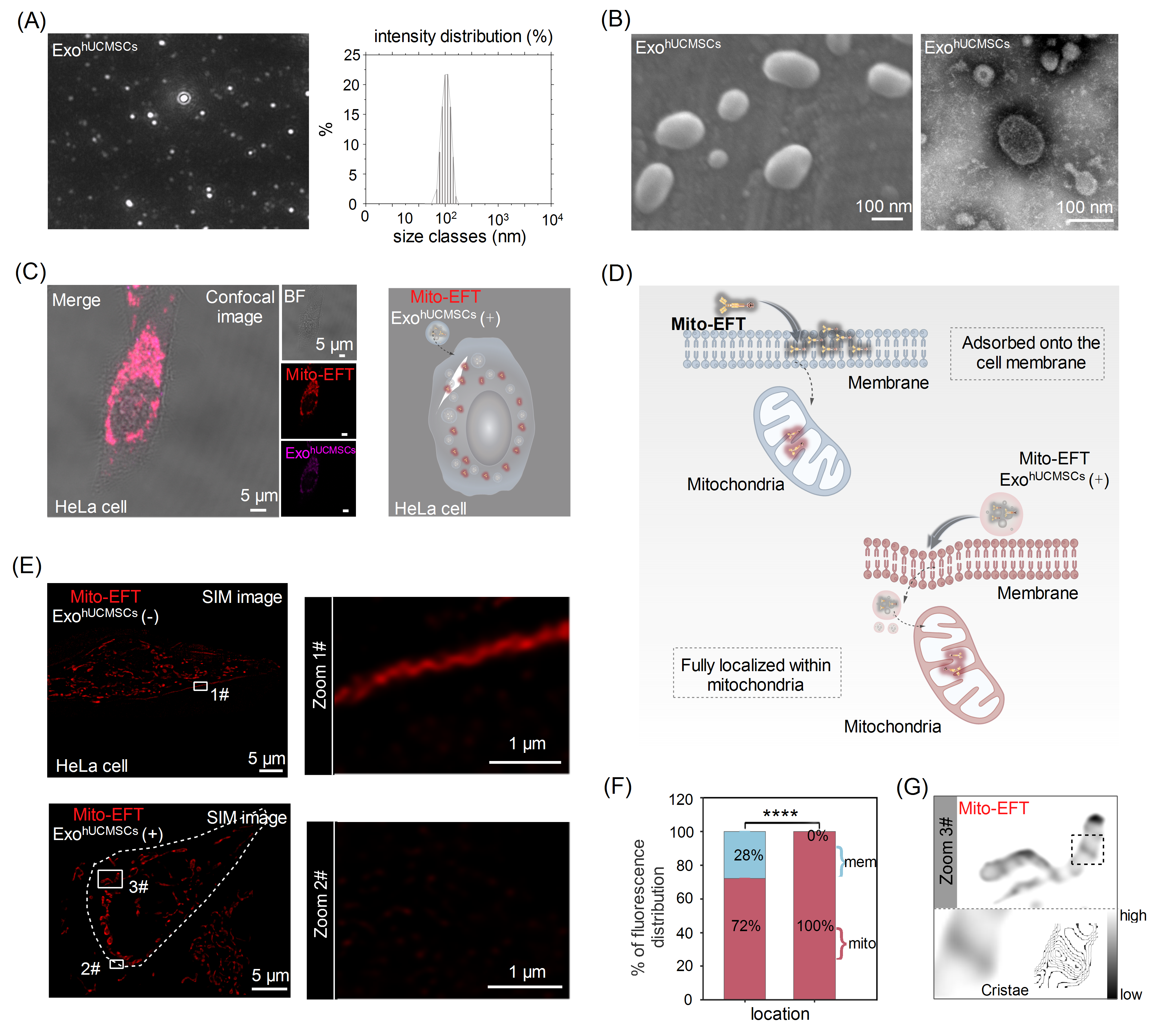
 Figure S7.** **Mito-EF was labeled by Mito-EFT probe in living cells.**

(A) The size distribution of Exo^hUCMSCs^ was analyzed by nanoparticle tracking analysis.

(B) Representative scanning electron microscope (left panel) and transmission electron microscopy (right panel) images of Exo^hUCMSCs^ (Scale bar, 100 nm).

(C) Dil‑labeled exosomes (purple fluorescence) and Mito-EFT (red fluorescence) were distributed in HeLa cells independently (Scale bar, 5 μm). The schematic representation indicates that the probe encapsulated in exosomes enters the cells.

(D) Schematic representation of Mito-EFT entering the cell and targeting mitochondria, when without encapsulation by Exo^hUCMSCs^, Mito-EFT partially resided on the cell membrane; when with encapsulation by Exo^hUCMSCs^, all Mito-EFT entered the cell and targeted the inner mitochondrial membrane. Created in BioRender. Shao, S. (2024) BioRender.com/q80r687

(E) Fluorescence images of HeLa cells incubated with Mito-EFT (1.0 μM) without or with Exo^hUCMSCs^ encapsulation for 30 min at 37°C (Scale bar, 5 μm); zoomed-in images within white rectangles depict the probe adsorbed onto the cell membrane (Scale bar, 1 μm).

(F)The statistical graph shows the fluorescence intensity distribution ratio of Mito-EFT at different positions. Exo^hUCMSCs^: Exosomes derived from hUCMSCs; hUCMSCs: human umbilical cord mesenchymal stem cells. Data are expressed as the mean ± SEM (*****P < 0.0001*).

(G) Representative images of mitochondria cristae using the Mito-EFT and local cristae magnification images and hot mapping.

Mito-EFT channel: Ex, 488 nm, Em, 600 nm - 650 nm.

**
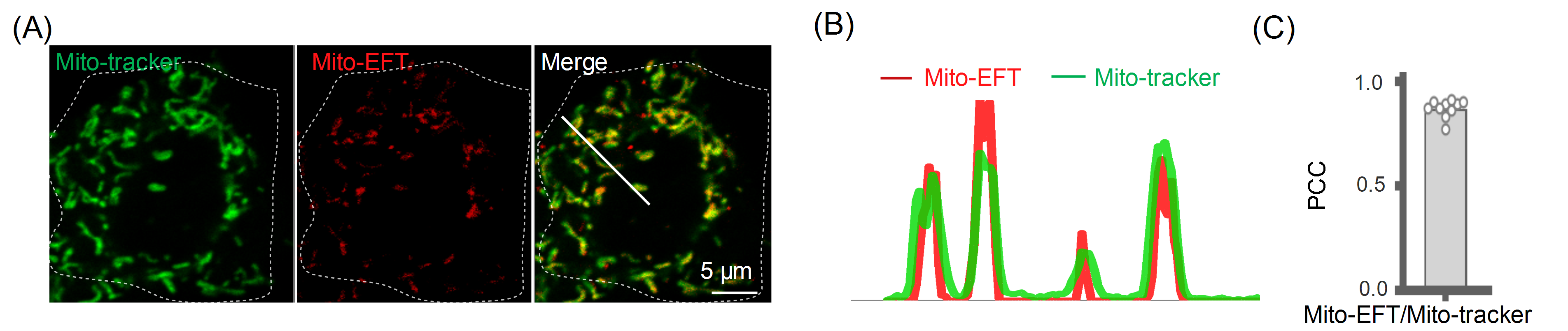
 Figure S8.** **Colocalization images of Mito-EFT (1.0 μM) with Mito-tracker in HeLa cells.**

(A) HeLa cells were co-stained with the commercial mitochondrial tracker, pKMTDR (100.0 nM), and Mito-EFT (1.0 μM) for 30 min at 37 °C (Scale bar, 5 μm). The white solid line indicated the region for fluorescence analysis as shown in panel (B).

(B) Fluorescence intensity profiles of Mito-EFT and Mito-tracker were measured in white lines from the images in panel (A).

(C) *The Pearson’s correlation coefficient* (PCC) value for Mito-EFT and Mito-tracker from panel (A).

Mito-EFT channel: Ex, 488 nm, Em, 600 nm - 650 nm; Mito-tracker channel: Ex, 640 nm; and Em, 641 nm - 694 nm.


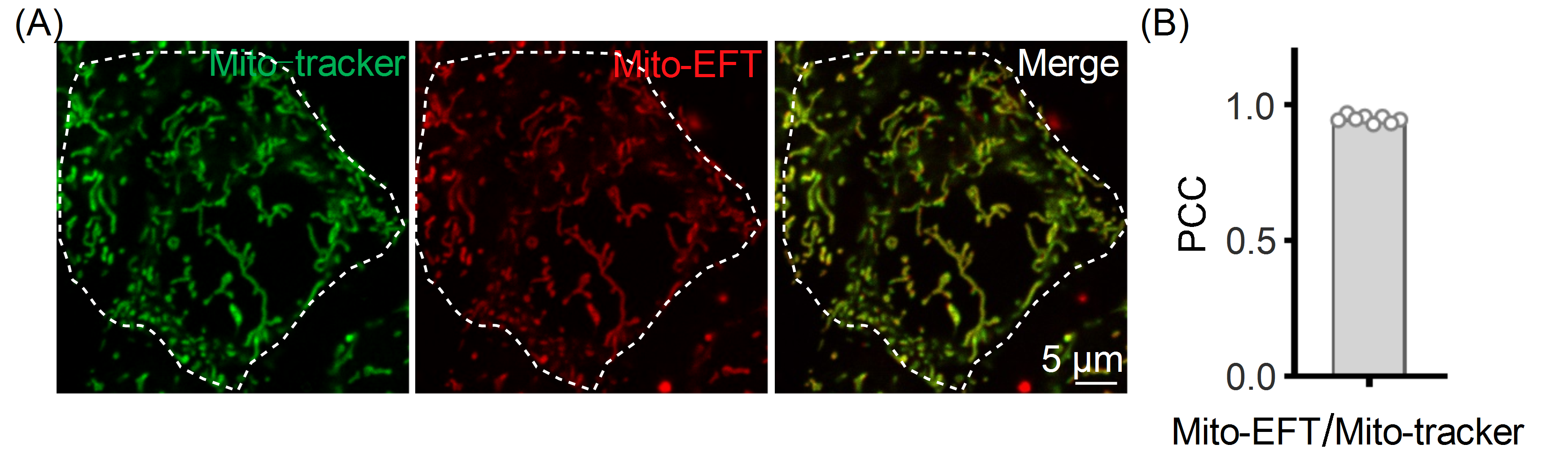


**Figure S9.** **Colocalization images of Mito-EFT (1.0 μM) with the commercial mitochondrial probe in HepG2 cells.**

(A) HepG2 cells co-stained with the commercial mitochondrial tracker, pKMTDR (100.0 nM), and Mito-EFT (1.0 μM) for 30 min at 37 °C (Scale bar, 5 μm).

(B) *The Pearson's correlation coefficient* (PCC) value for Mito-EFT and Mito-tracker from (A).

Mito-EFT channel: Ex, 488 nm, Em, 600 - 650 nm; Mito-tracker channel: Ex, 647 nm; Em, 641 - 694 nm.


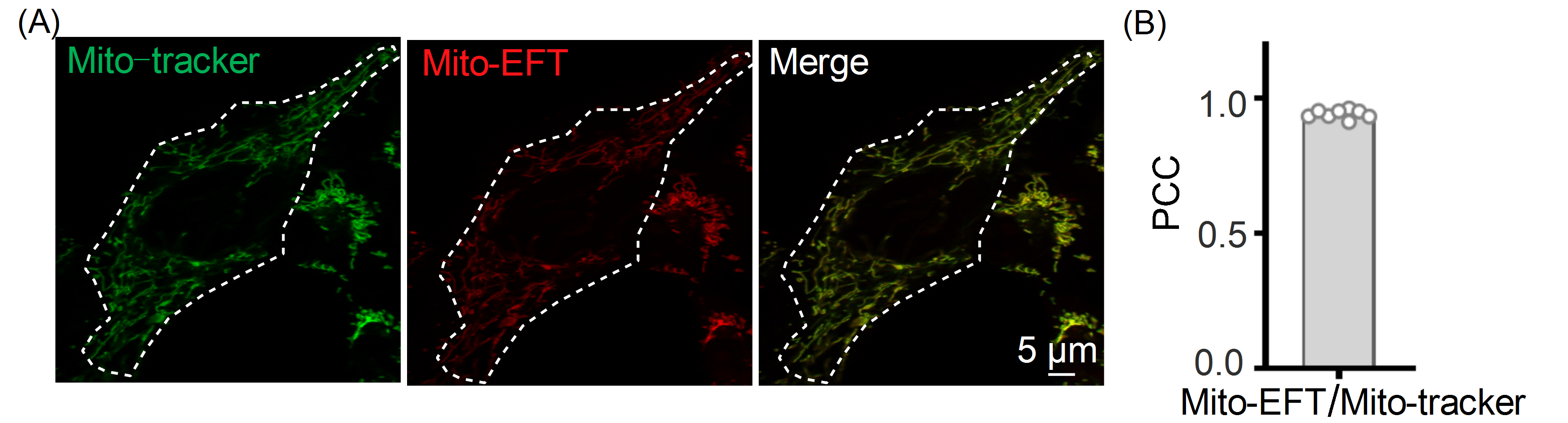


**Figure S10. Colocalization images of Mito-EFT with the commercial mitochondria probe in human skeletal muscle cells HSkM.**

(A) HSkM cells co-stained with the commercial mitochondrial tracker, pKMTDR (100.0 nM), and Mito-EFT (1.0 μM) for 30 min at 37 °C (Scale bar, 5 μm).

(B)*The Pearson's correlation coefficient* (PCC) value for Mito-EFT and Mito-tracker from (A).

Mito-EFT channel: Ex, 488 nm, Em, 600 - 650 nm; Mito-tracker channel: Ex, 647 nm; Em, 641 - 694 nm.


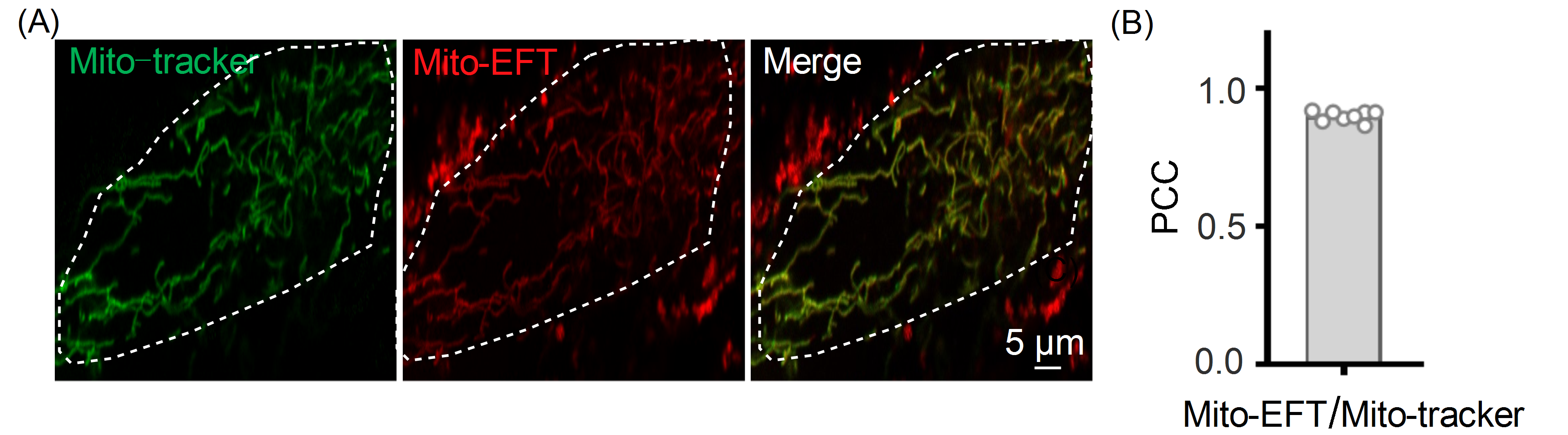


**Figure S11. Colocalization images of Mito-EFT with the commercial mitochondria probe in senescent HSkM cells.**

(A) Senescent HSkM cells co-stained with the commercial mitochondrial tracker, pKMTDR (100.0 nM), and Mito-EFT (1.0 μM) for 30 min at 37 ℃ (Scale bar, 5 μm).

(B) *The Pearson**'s correlation coefficient* (PCC) value for Mito-EFT and Mito-tracker from (A).

Mito-EFT channel: Ex, 488 nm, Em, 600 - 650 nm; Mito-tracker channel: Ex, 647 nm; Em, 641 - 694 nm.


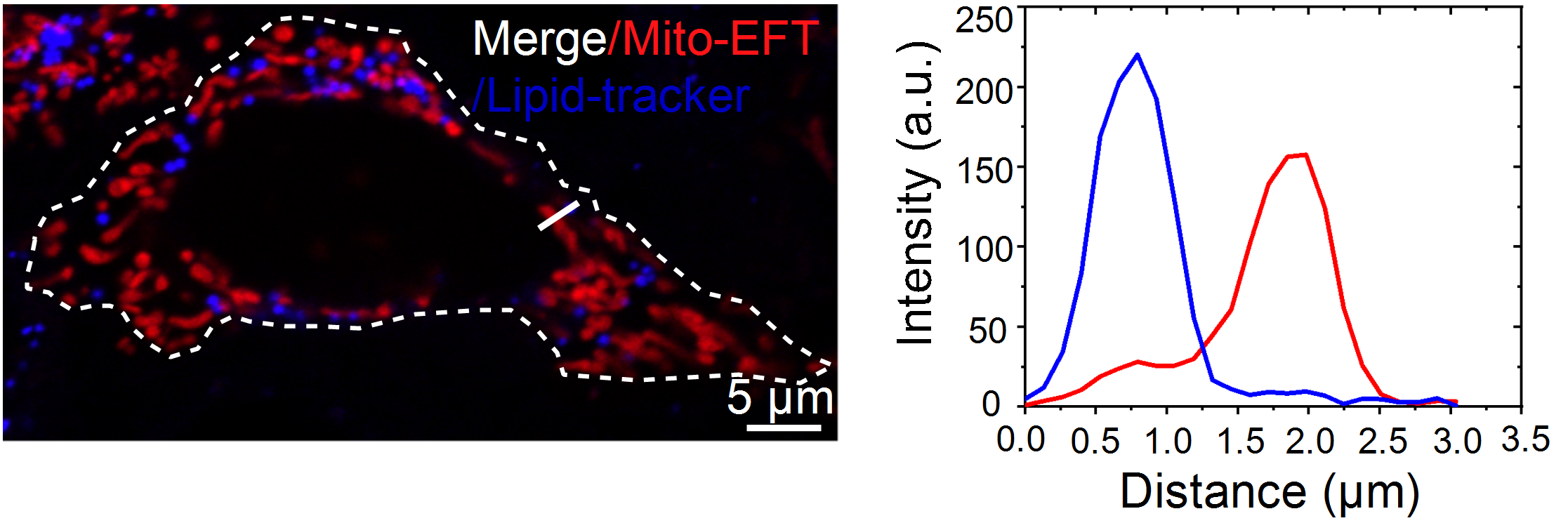


**Figure S12.** **Colocalization images of Mito-EFT (1.0 μM) with lipid droplet in HeLa cells.**

HeLa cells co-stained with Mito-EFT (1.0 μM) and lipid droplet commercial dye (LDB, 200.0 nM) and their fluorescence intensity of the distance between different organelles (Scale bar, 5 μm). Mito-EFT channel: Ex, 488 nm, Em, 600 - 650 nm; Lipid-Tracker channel: Ex, 405 nm, Em, 420 - 450 nm.


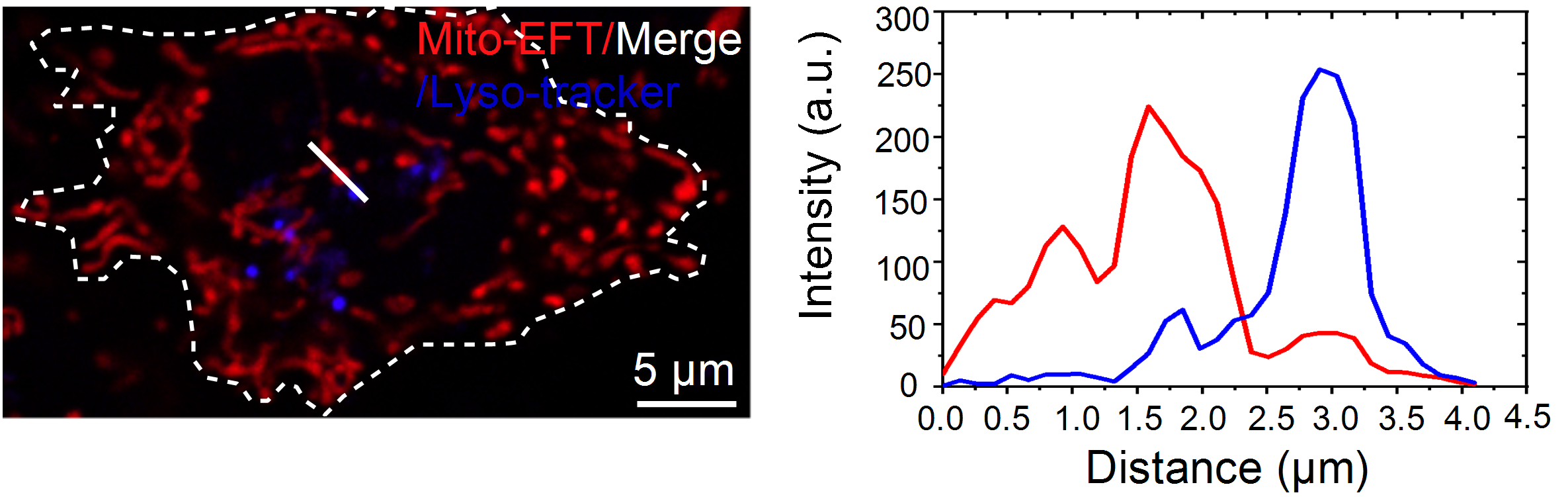


**Figure S13.** **Colocalization images of Mito-EFT (1.0 μM) with lysosome in HeLa cells.**

HeLa cells co-stained with Mito-EFT (1.0 μM) and Lyso-Tracker Blue (LTB, 200.0 nM) and their fluorescence intensity of the distance between different organelles (Scale bar, 5 μm). Mito-EFT channel: Ex, 488 nm, Em, 600 - 650 nm; Lyso-Tracker channel: Ex, 405 nm, Em, 420 - 450 nm.


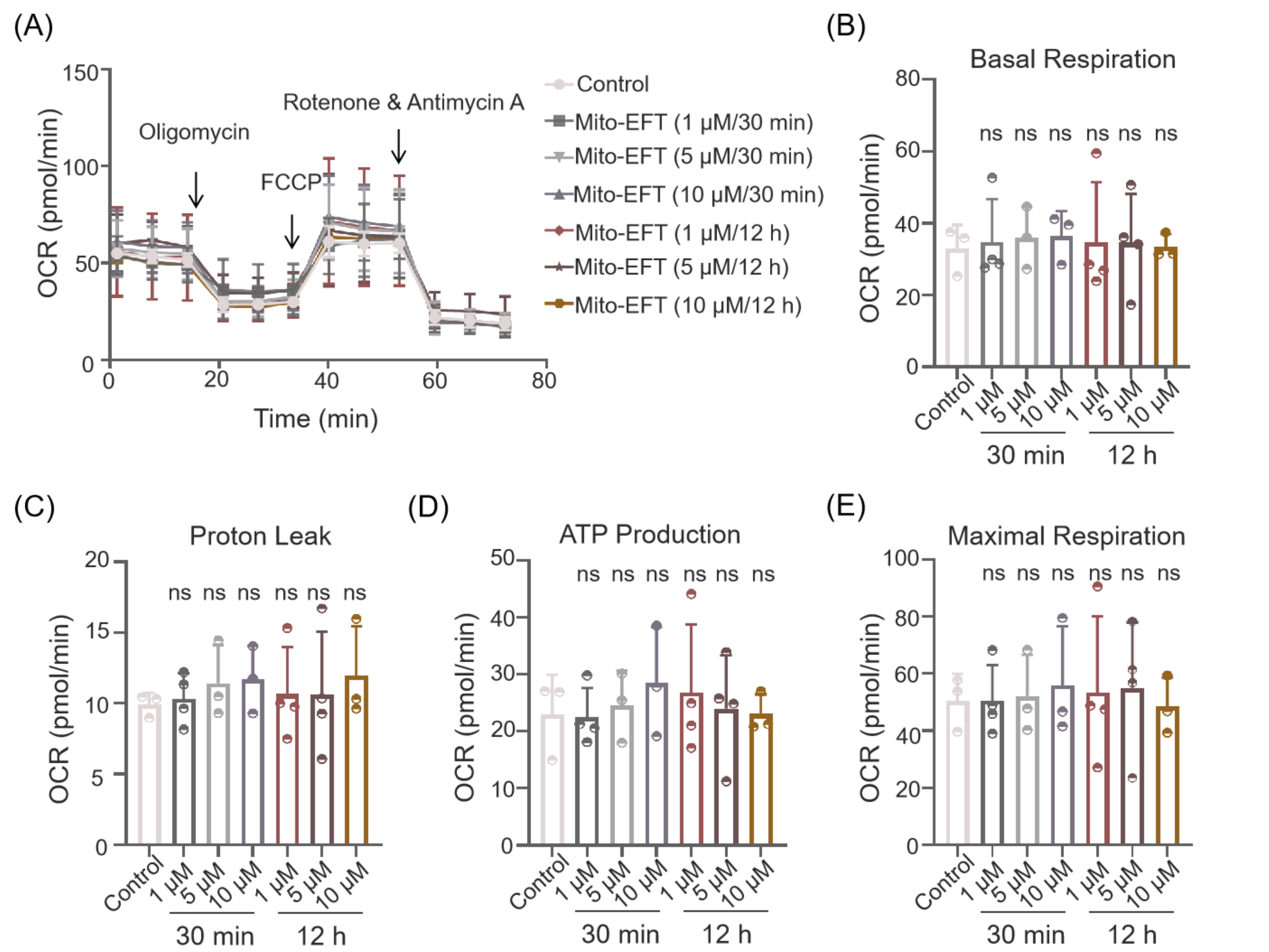


**Figure S14.** **The** **mitochondrial respiratory function was examined using the OCR assay.**

(A) The oxygen consumption rate (OCR) was detected as an indicator for oxidative phosphorylation (OXPHOS).

(B-E) HeLa cells treated with Mito-EFT with different concentrations and times for (B) mitochondrial oxidative phosphorylation and ATP synthesis, peak oxygen consumption, (C) proton leak, (D) ATP production, and (E) maximal respiration. Data were expressed as the mean ± SEM (n = 3, n.s.).


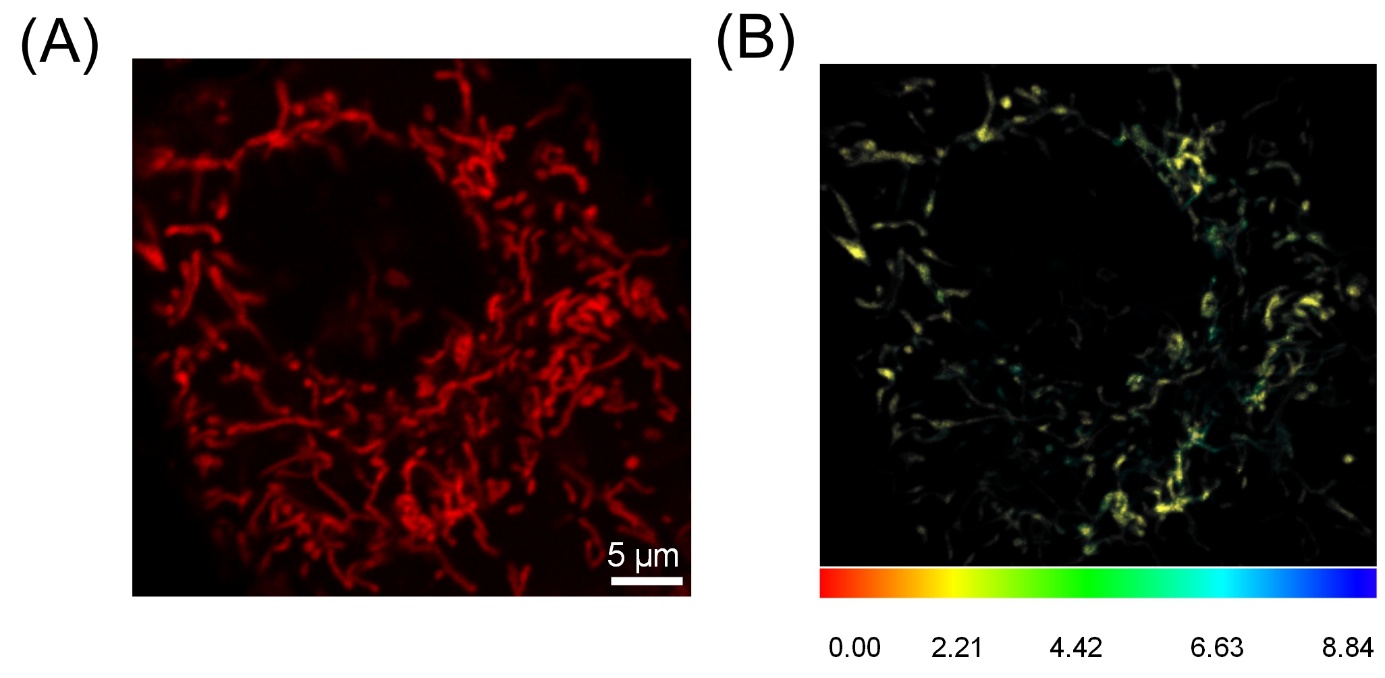


**Figure S15.** **Confocal images of Mito-EFT (1.0 μM)** **in HeLa cells.**

(A) Confocal image of HeLa cells stained with Mito-EFT (1.0 μM) for 30 min (Scale bar, 5 μm). Mito-EFT channel: Ex, 488 nm, Em, 600 - 650 nm.

(B) 3D distribution map of Mito-EFT (1.0 μM) in HeLa cells.

###
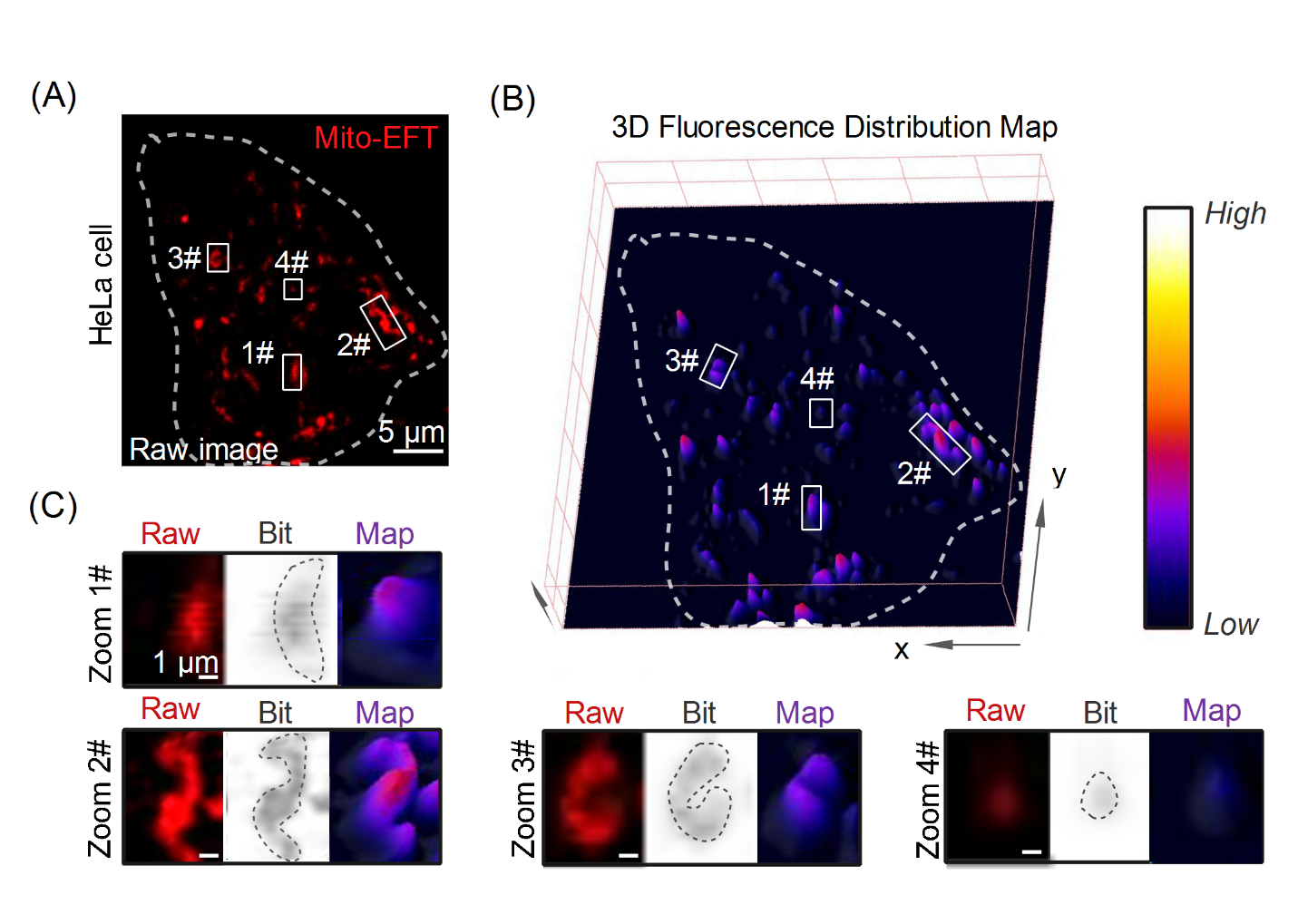


**Figure S16. Characterization of Mito-EFT in living cells.**

1. HeLa cells were incubated with Mito-EFT (1.0 μM) for 30 min at 37 °C (Scale bar, 5 μm).
2. 3D surface fluorescence distribution map of HeLa cells incubated with Mito-EFT.

(C) Zoom-in images of white rectangles and fluorescence intensity heat map of Mito-EFT from (A, B) (Scale bar, 1 μm). Mito-EFT channel: Ex, 488 nm, Em, 600 - 650 nm.


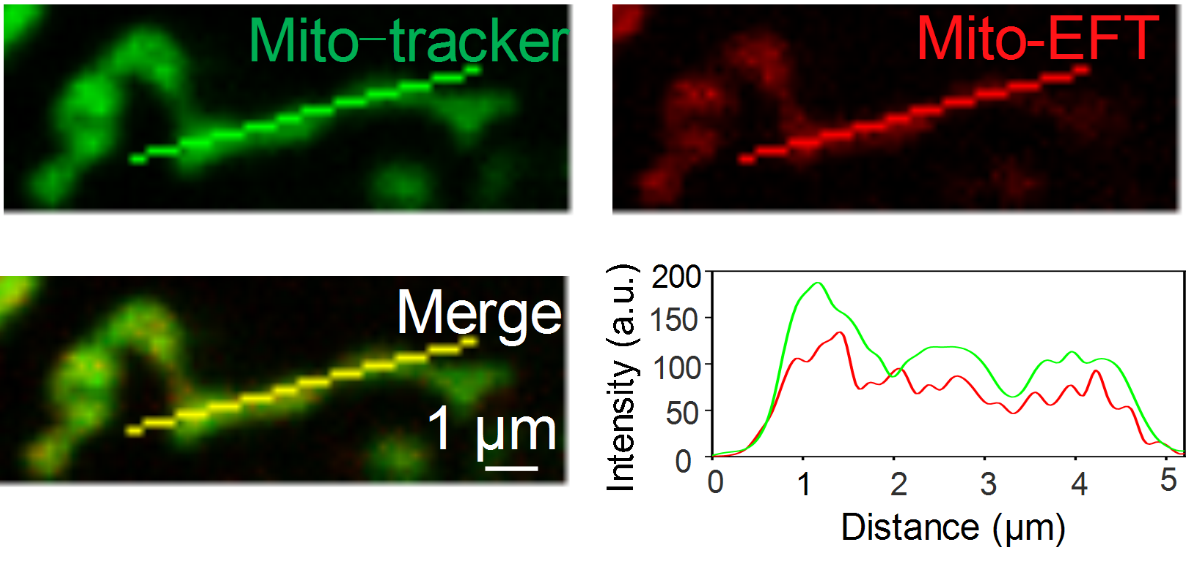


**Figure S17.** **Colocalization images of Mito-EFT (1.0 μM) with Mito-tracker in fiber-like mitochondria.**

Mito-EFT (1.0 μM) tracking of fiber-like mitochondria was co-localized with a commercial mitochondrial probe (Scale bar, 1 μm). Mito-EFT channel: Ex, 488 nm, Em, 600 - 650 nm; Mito-tracker channel: Ex, 647 nm; Em, 641 - 694 nm.


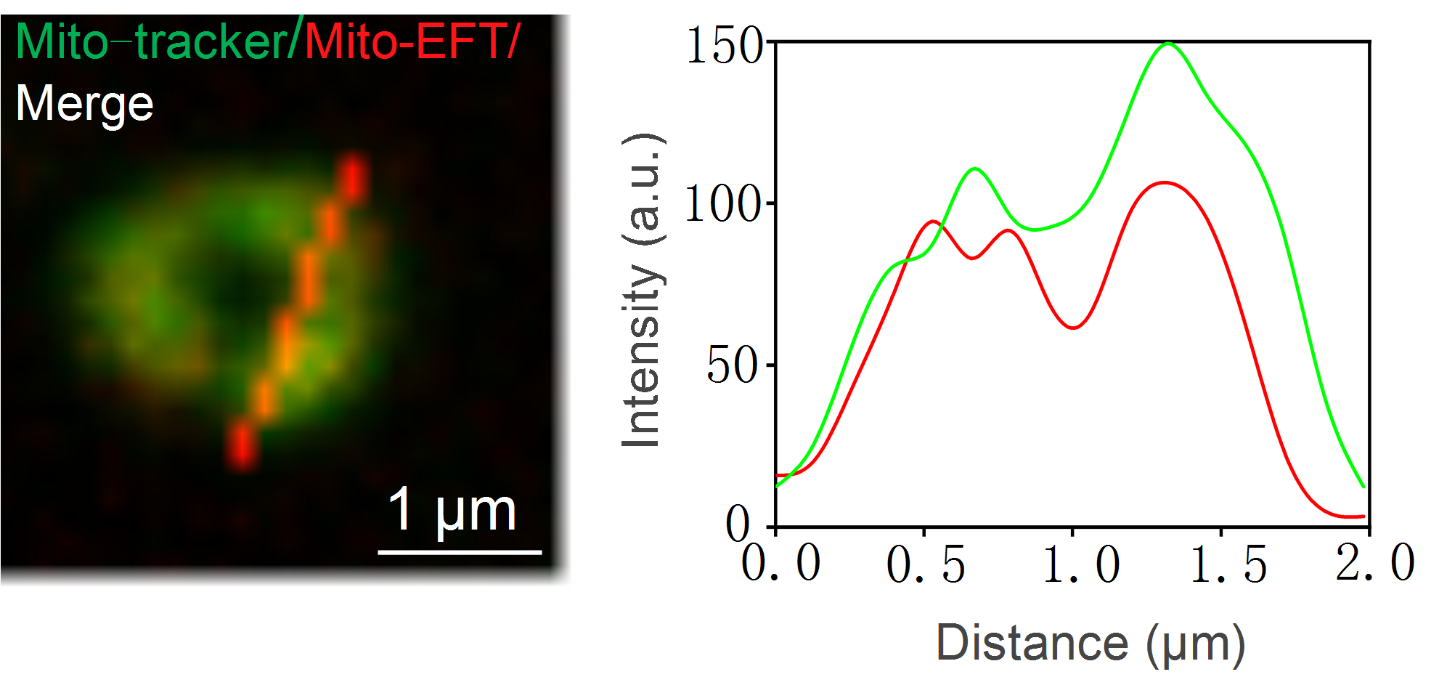


**Figure S18. Colocalization images of Mito-EFT (1.0 μM) with Mito-tracker in donut-like mitochondria.**

Mito-EFT (1.0 μM) tracking of donut-like mitochondria was co-localized with a commercial mitochondrial probe (Scale bar, 1 μm). Mito-EFT channel: Ex, 488 nm, Em, 600 - -650 nm; Mito-tracker channel: Ex, 647 nm; Em, 641 - 694 nm.


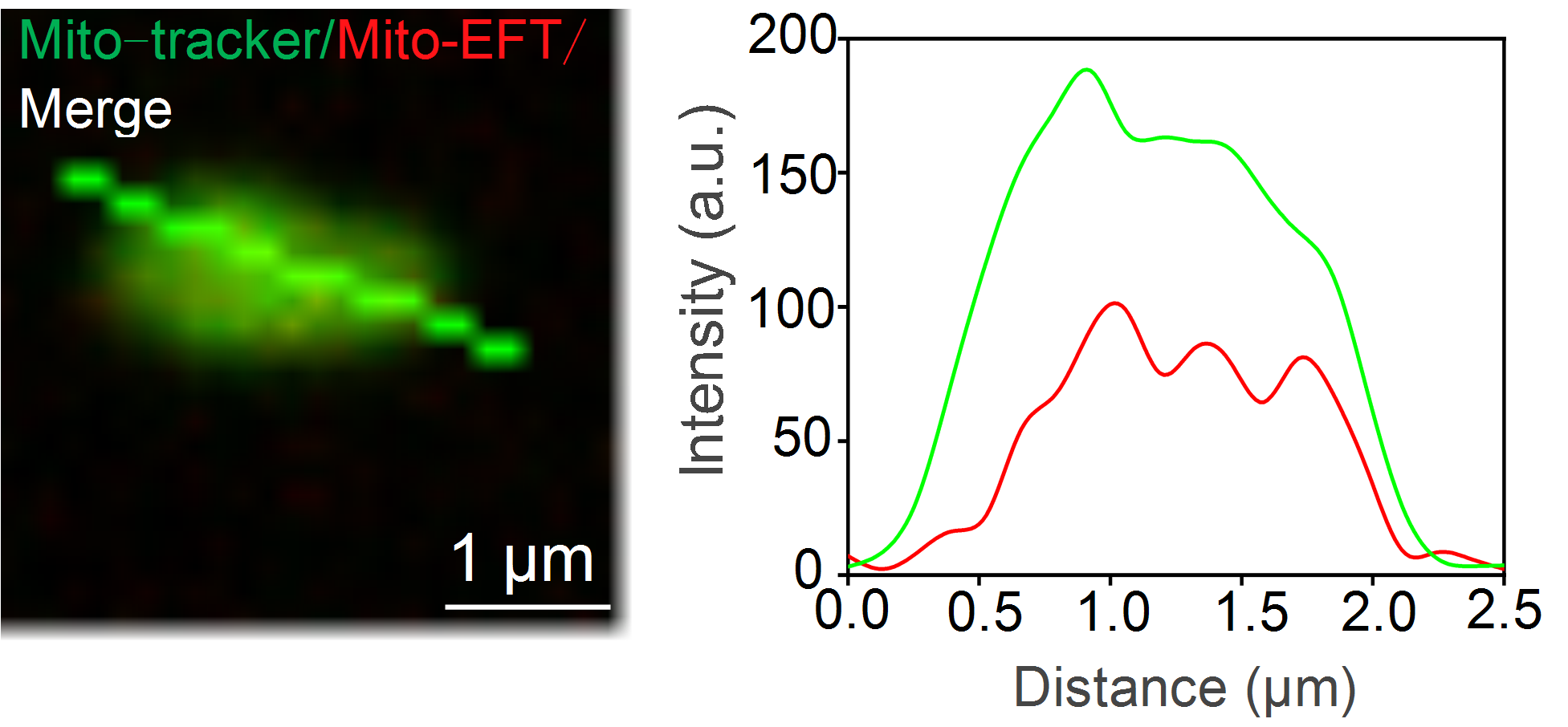


**Figure S19. Colocalization images of Mito-EFT (1.0 μM) with Mito-tracker in round-like mitochondria.**

Mito-EFT (1.0 μM) tracking of round-like mitochondria was co-localized with a commercial mitochondrial probe (Scale bar, 1 μm). Mito-EFT channel: Ex, 488 nm, Em, 600 - 650 nm; Mito-tracker channel: Ex, 647 nm; Em, 641 - 694 nm.


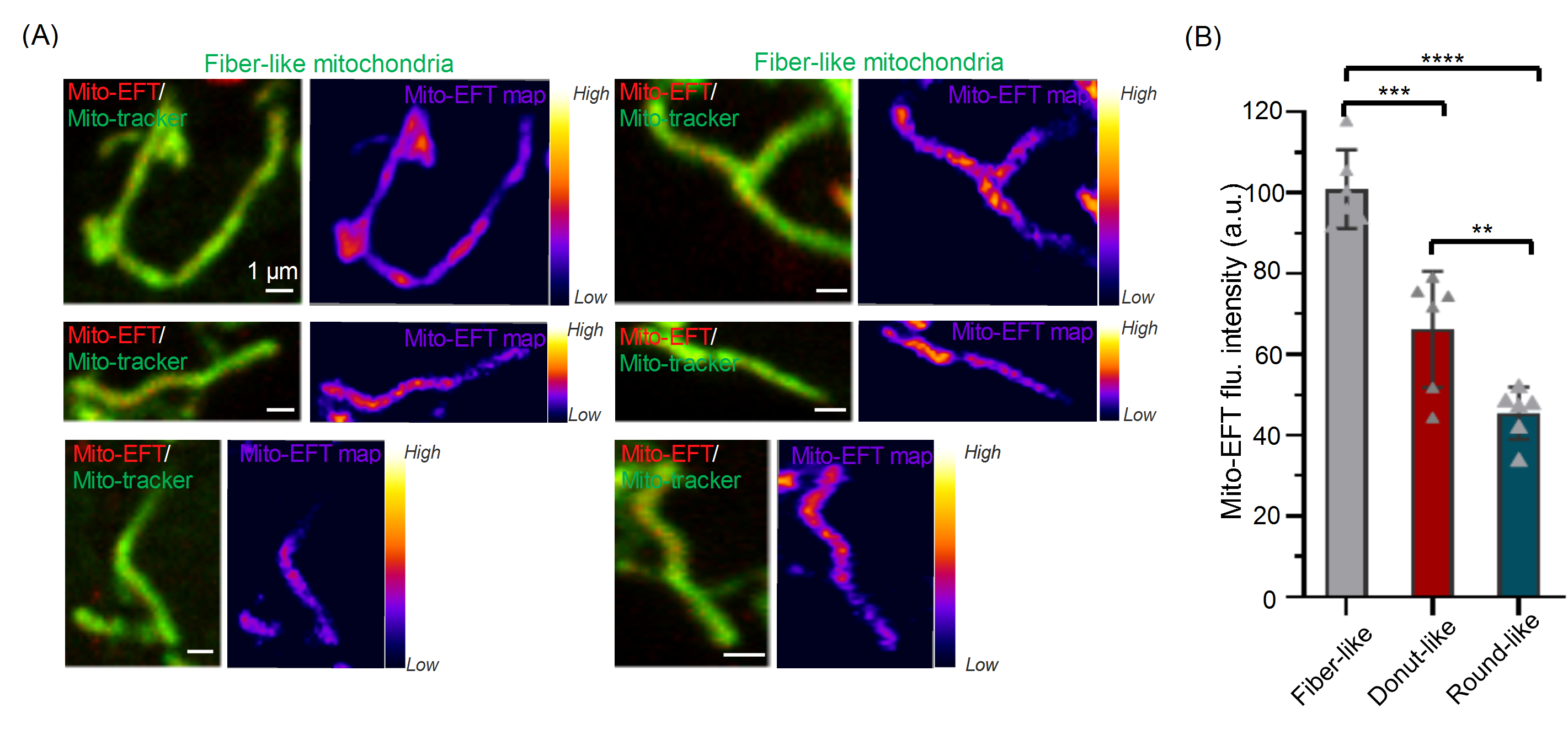


**Figure S20. Mito-EFT tracking of fiber-like mitochondrial morphology.**

(A)Mito-EFT and Mito-tracker tracking of fiber-like mitochondria morphology (Scale bar, 1 μm).

(B) Quantitative analysis of the Mito-EFT fluorescent intensity distribution in different forms of mitochondria.Data were expressed as the mean ± SEM (n = 6, ***P < 0.01, ***P < 0.001, ****P < 0.0001*).

Mito-EFT channel: Ex, 488 nm, Em, 600 - 650 nm; Mito-tracker channel: Ex, 647 nm; Em, 641 - 694 nm.


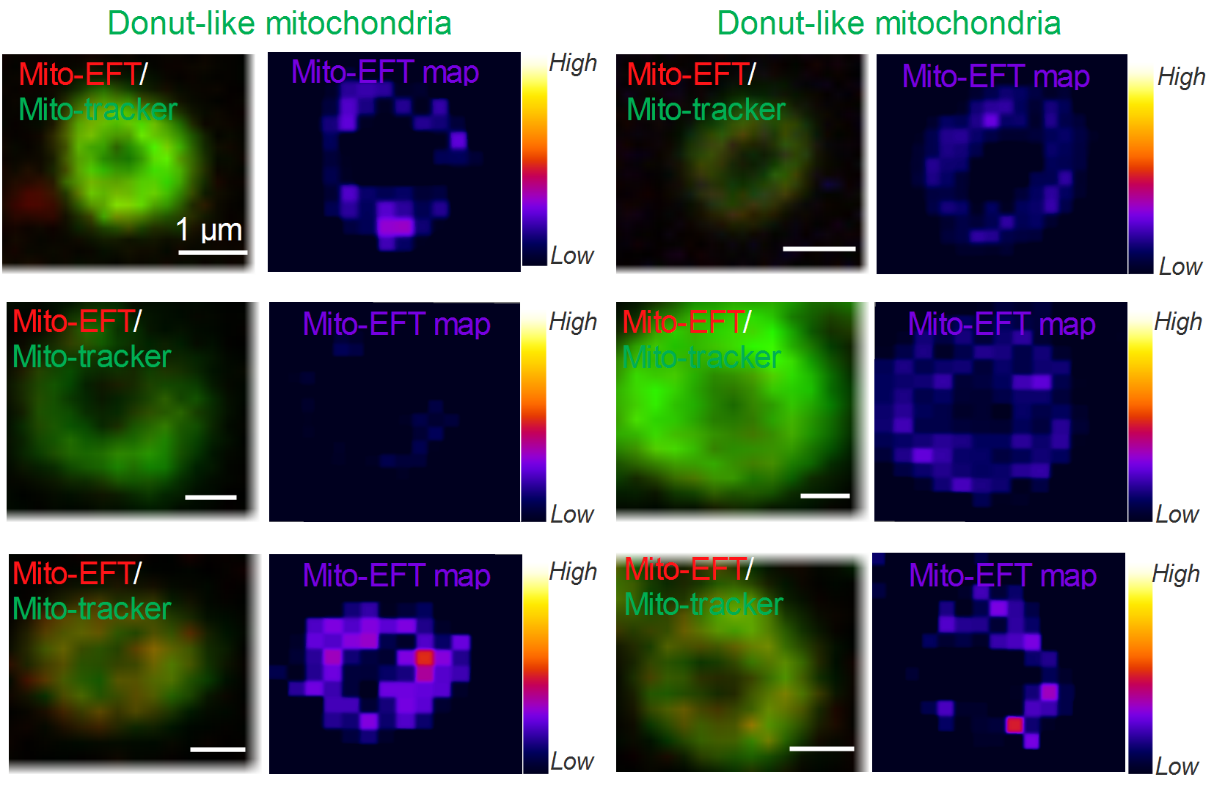


**Figure S21.** **Mito-EFT tracking of donut-like mitochondrial morphology.**

Mito-EFT and Mito-tracker tracking of donut-like mitochondria morphology (Scale bar, 1 μm). Mito-EFT channel: Ex, 488 nm, Em, 600 - 650 nm; Mito-tracker channel: Ex, 647 nm; Em, 641 - 694 nm.


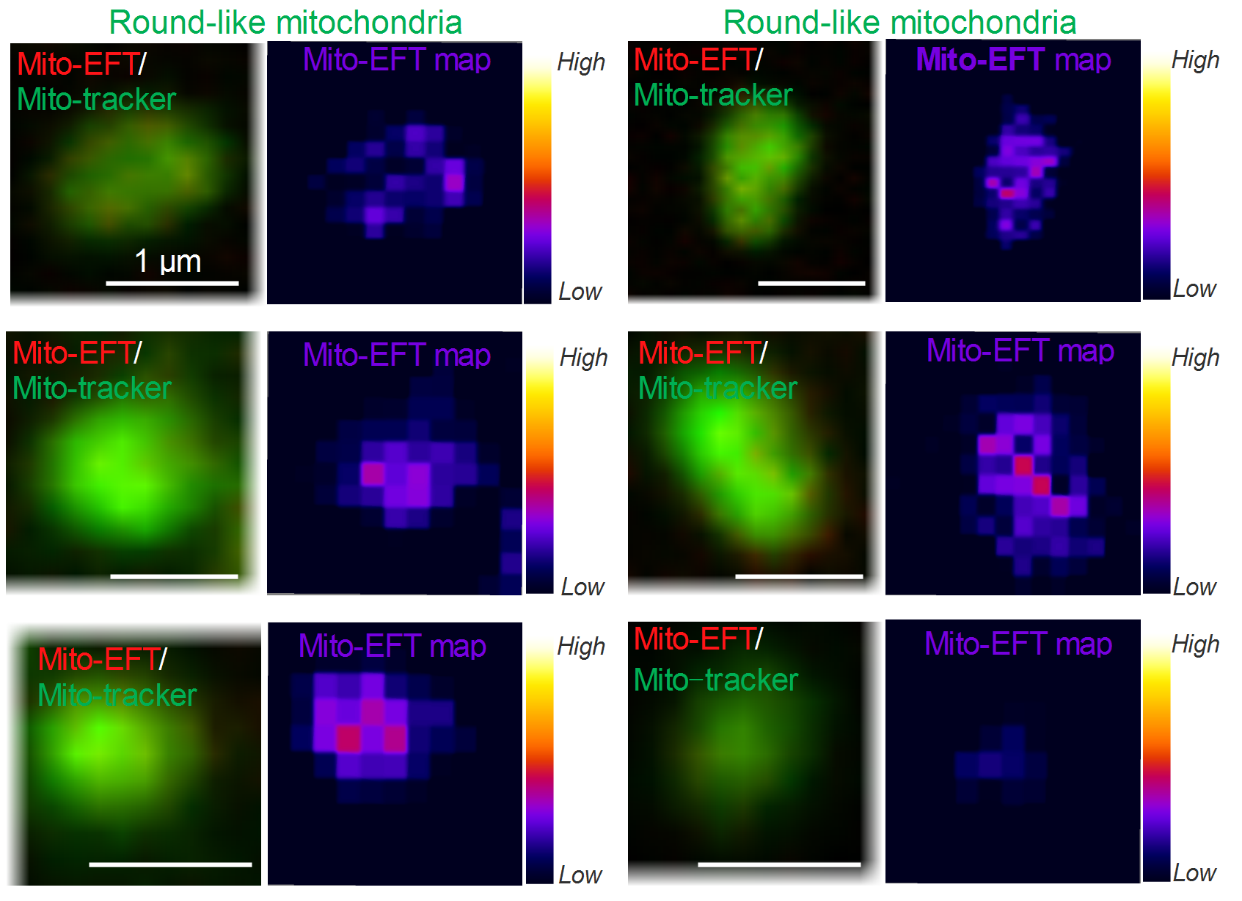


**Figure S22.** **Mito-EFT tracking of round-like mitochondrial morphology.**

Mito-EFT and Mito-tracker tracking of round-like mitochondria morphology (Scale bar, 1 μm). Mito-EFT channel: Ex, 488 nm, Em, 600 - 650 nm; Mito-tracker channel: Ex, 647 nm; Em, 641 - 694 nm.


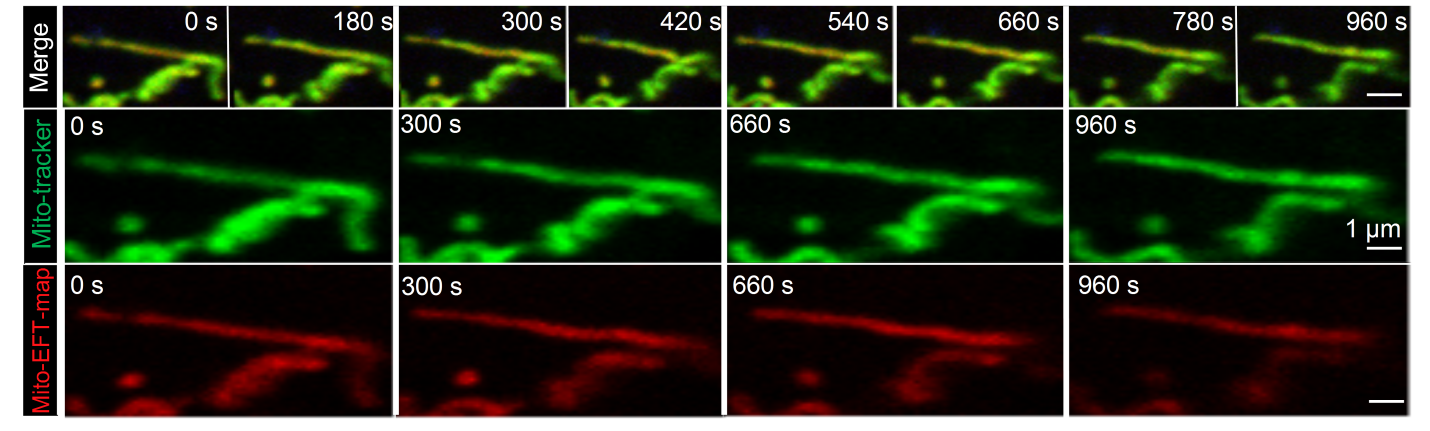


**Figure S23. Long-term imaging of the Mito-EFT.**

The dynamic process of mitochondria in HeLa cells stained with Mito-EFT. The time interval between each frame was ~ 60 s (Scale bar, 1 μm).

Mito-EFT channel: Ex, 488 nm, Em, 600 - 650 nm; Mito-tracker channel: Ex, 647 nm; Em, 641 - 694 nm.


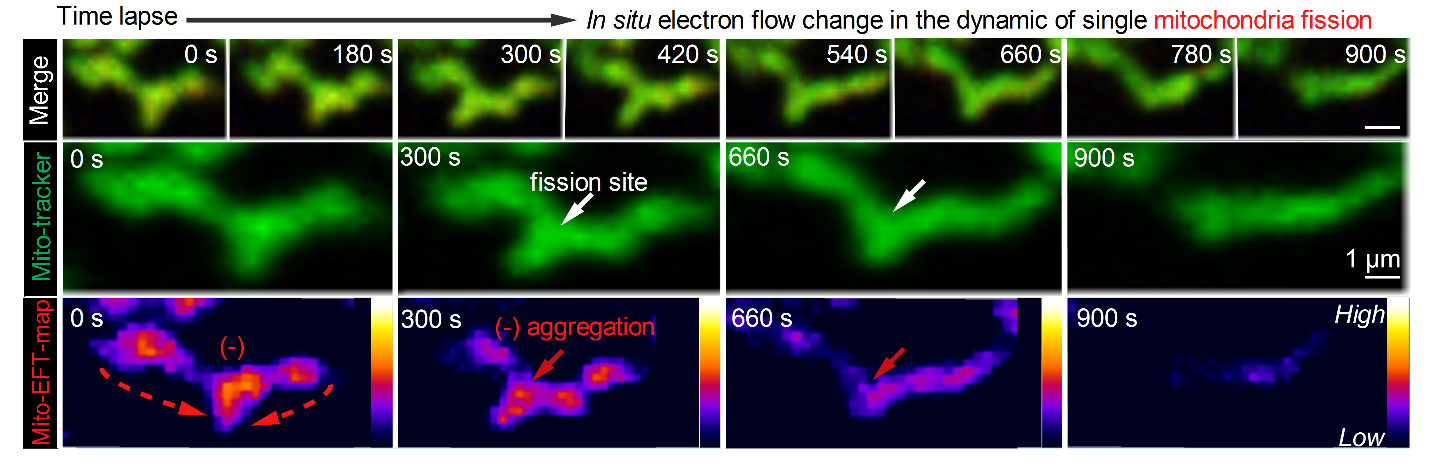


**Figure S24.** **Long-term imaging of the Mito-tracker and Mito-EFT.**

The dynamic process of mitochondrial fission in HeLa cells stained with Mito-EFT. Red arrows indicate Mito-EF aggregation in the representative mitochondrial fission event. The time interval between each frame was ~ 60 s (Scale bar, 1 μm). Mito-EFT channel: Ex, 488 nm, Em, 600 - 650 nm; Mito-tracker channel: Ex, 647 nm; Em, 641 - 694 nm.

**
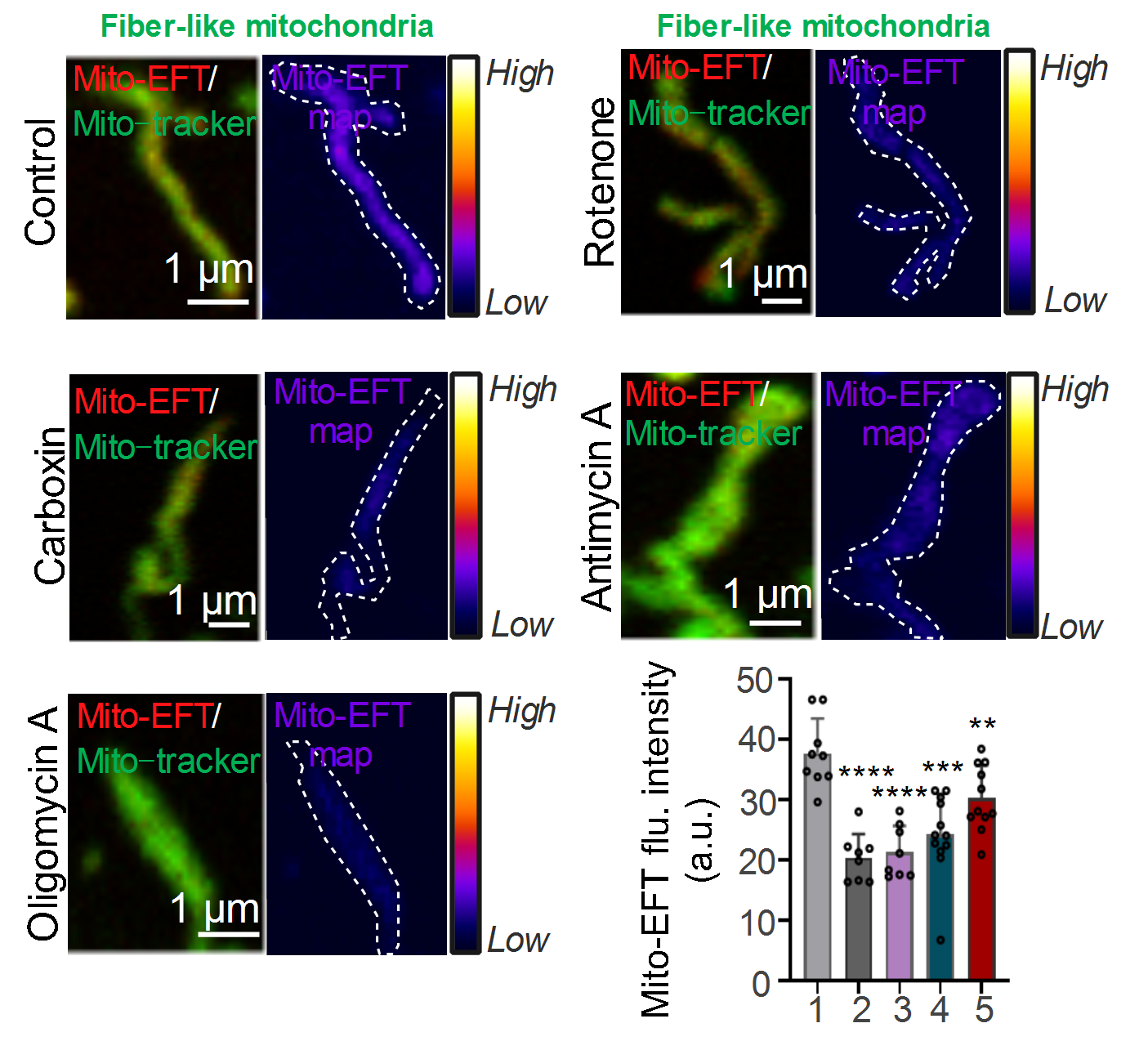
**

**Figure S25.** **The fluorescence intensity changes of Mito-EFT in the tracking of fiber-like mitochondria treated with OXPHOS inhibitors.**

Mito-EFT and Mito-tracker tracking of fiber-like mitochondria treated with OXPHOS inhibitors and quantitative analysis of the Mito-EFT fluorescent intensity distribution (Scale bar, 1 μm). 1. Untreated; 2. Rotenone; 3. Carboxin; 4. Antimycin A; and 5. Oligomycin A. Data were expressed as the mean ± SEM (n = 10 cells, ***P < 0.01, ***P < 0.001, ****P < 0.0001*). Mito-EFT channel: Ex, 488 nm, Em, 600 - 650 nm. Mito-tracker channel: Ex, 647 nm; Em, 641 - 694 nm.


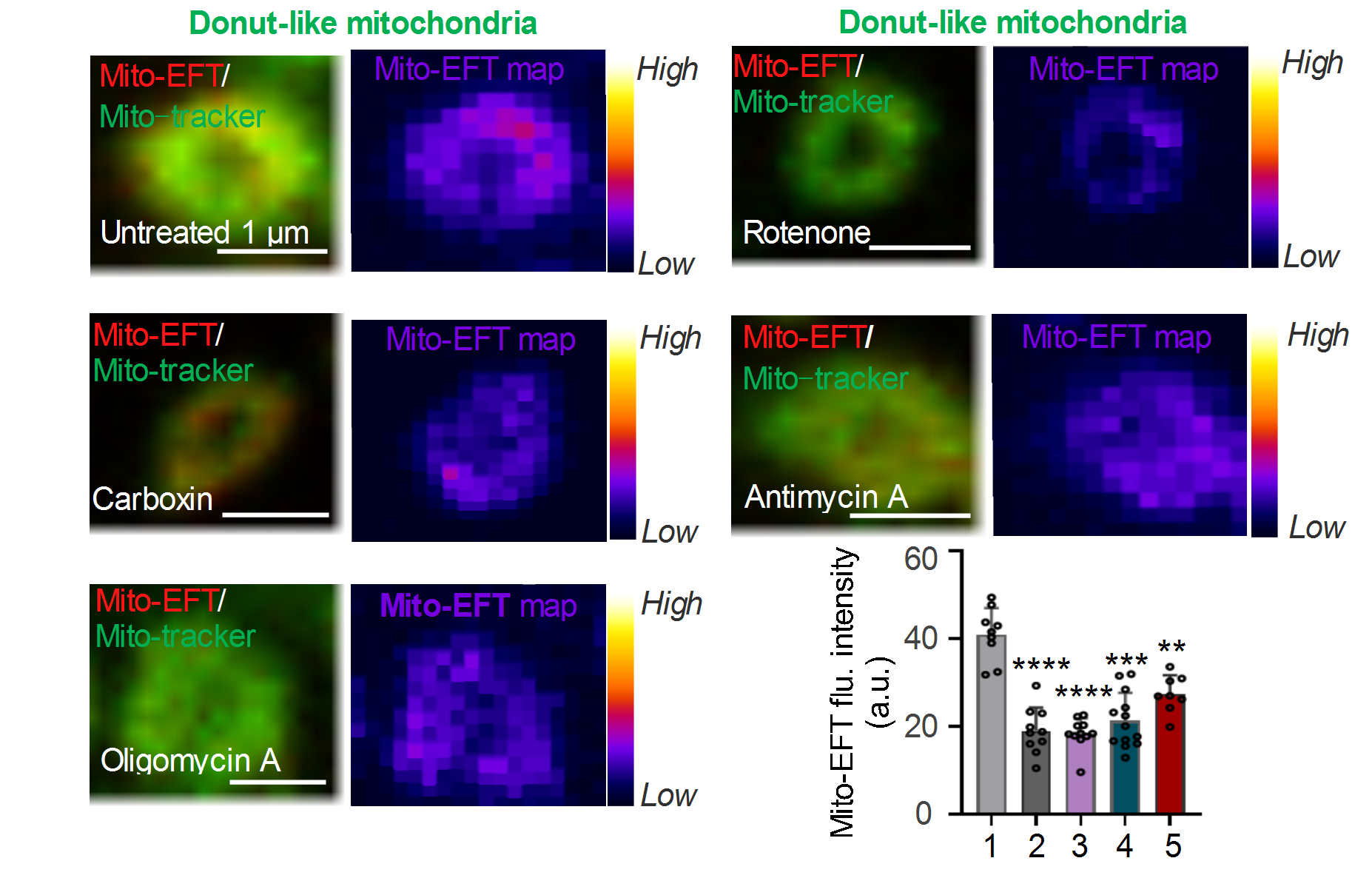


**Figure S26. The fluorescence intensity changes of Mito-EFT in the tracking of donut-like mitochondria treated with OXPHOS inhibitors.**

Mito-EFT and Mito-tracker tracking of donut-like mitochondria treated with OXPHOS inhibitors and quantitative analysis of the Mito-EFT fluorescent intensity distribution (Scale bar, 1 μm). 1. Untreated; 2. Rotenone; 3. Carboxin; 4. Antimycin A; and 5. Oligomycin A. Data were expressed as the mean ± SEM (n = 8, ***P < 0.01, ***P < 0.001, ****P < 0.0001*). Mito-EFT channel: Ex, 488 nm, Em, 600 - 650 nm. Mito-tracker channel: Ex, 647 nm; Em, 641 - 694 nm.


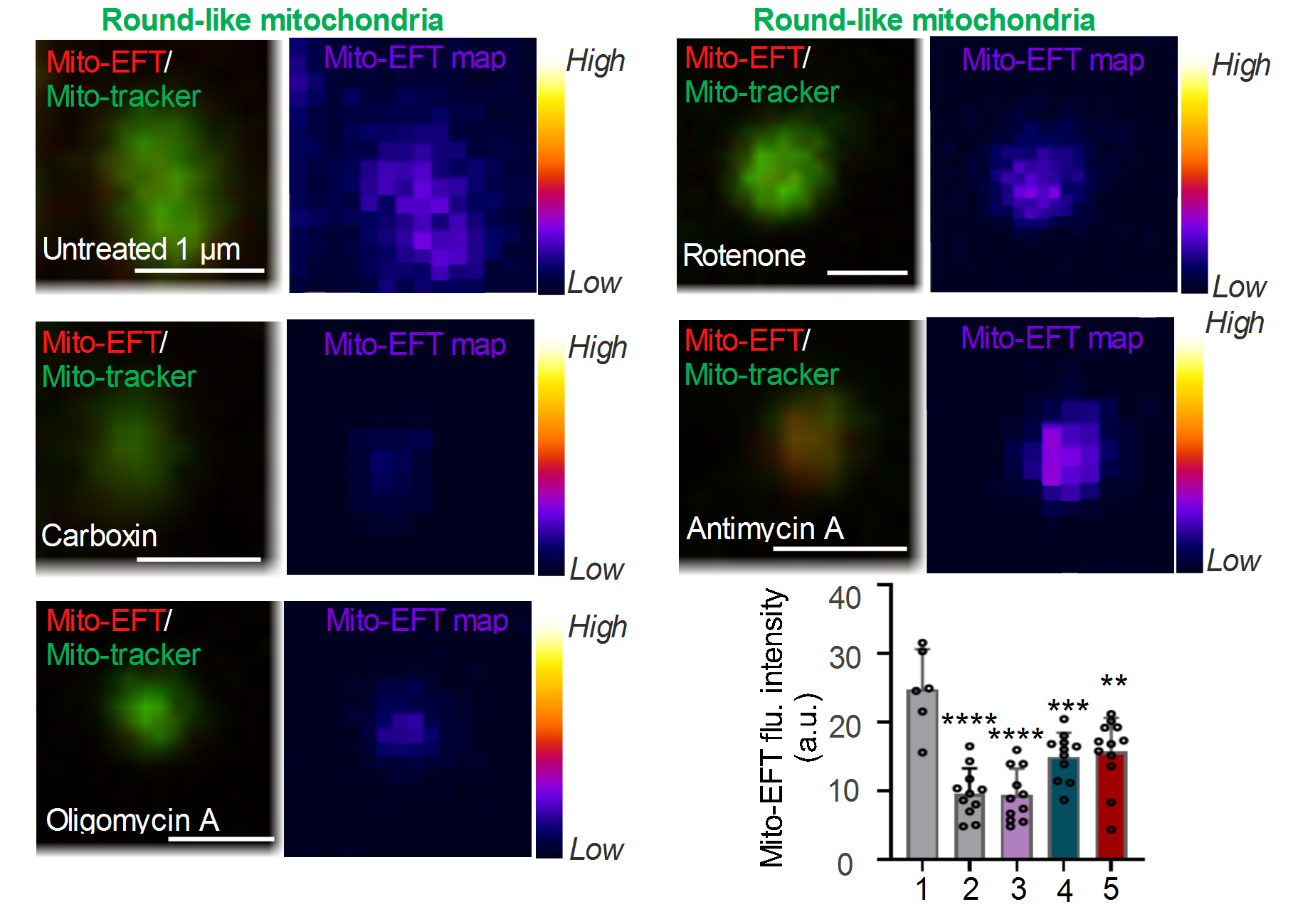


**Figure S27.** **The fluorescence intensity changes of Mito-EFT in the tracking of round-like mitochondria treated with OXPHOS inhibitors.**

Mito-EFT and Mito-tracker tracking of round-like mitochondria treated with OXPHOS inhibitors and quantitative analysis of the Mito-EFT fluorescent intensity distribution (Scale bar, 1 μm). 1. Untreated; 2. Rotenone; 3. Carboxin; 4. Antimycin A; and 5. Oligomycin A. Data were expressed as the mean ± SEM (n = 10 cells, ***P < 0.01, ***P < 0.001, ****P < 0.0001*). Mito-EFT channel: Ex, 488 nm, Em, 600 - 650 nm. Mito-tracker channel: Ex, 647 nm; Em, 641 - 694 nm.


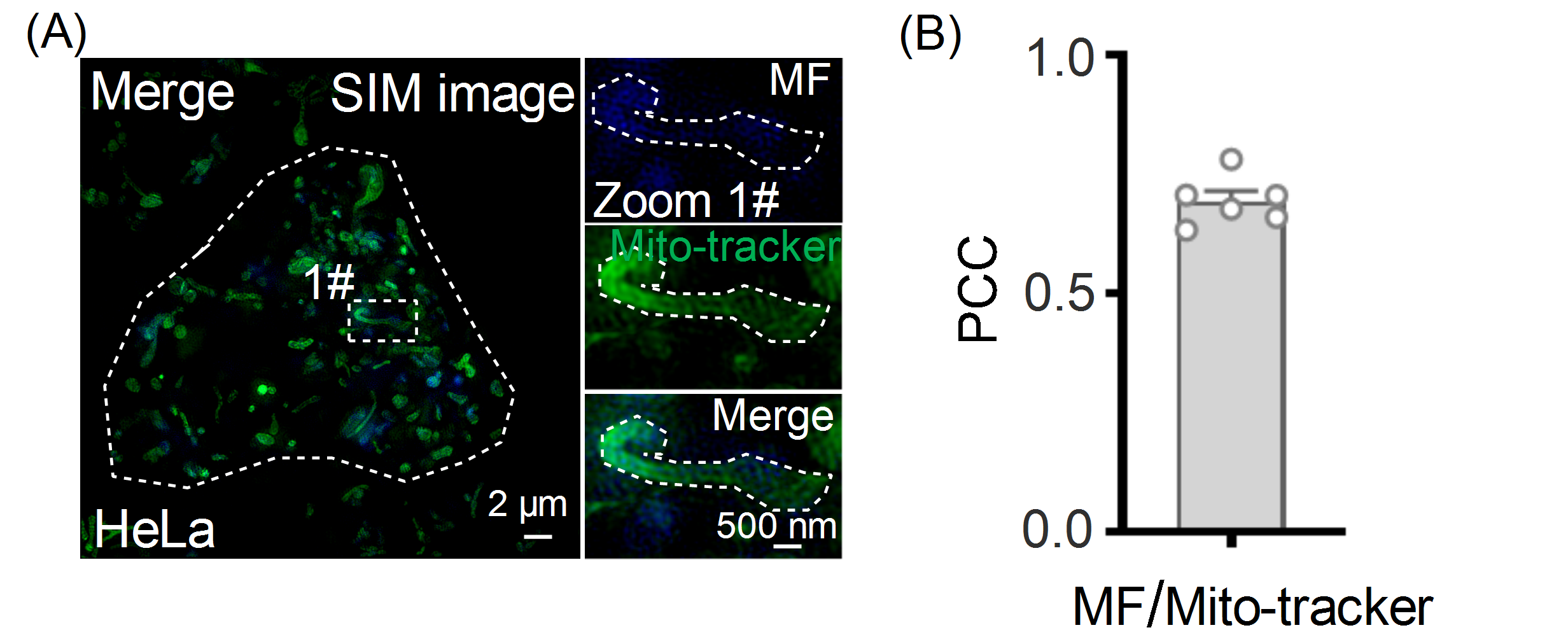


**Figure S28.** **Colocalization images of MF (10.0 μM) with the commercial mitochondrial probe in HeLa cells.**

(A) HeLa cells co-stained with the commercial mitochondrial tracker, pKMTDR (100.0 nM), and MF (10.0 μM) for 30 min at 37 °C (Scale bar, 2 μm). Zoomed-in images were of white rectangles #1 indicating Mito-tracker and MF tracking mitochondria (Scale bar, 500 nm); MF: Magnoflorine.

(B) *The Pearson's correlation coefficient* (PCC) value for MF and Mito-tracker from (A).

MF channel: Ex, 405 nm, Em, 411 - 553 nm; Mito-tracker channel: Ex, 647 nm; Em, 641 - 694 nm.
